# Using ordinal outcomes to construct and select biomarker combinations for single-level prediction

**DOI:** 10.1186/s41512-018-0028-3

**Published:** 2018-05-21

**Authors:** Allison Meisner, Chirag R. Parikh, Kathleen F. Kerr

**Affiliations:** 1 Department of Biostatistics, Johns Hopkins Bloomberg School of Public Health, Baltimore, MD USA; 2 Program of Applied Translational Research, Department of Medicine, Yale School of Medicine, New Haven, CT USA; 3 Department of Internal Medicine, Veterans Affairs Medical Center, West Haven, CT USA; 4 Department of Biostatistics, University of Washington, Seattle, WA USA

**Keywords:** Biomarker, Combinations, Ordinal

## Abstract

**Background:**

Biomarker studies may involve an ordinal outcome, such as no, mild, or severe disease. There is often interest in predicting one particular level of the outcome due to its clinical significance.

**Methods:**

A simple approach to constructing biomarker combinations in this context involves dichotomizing the outcome and using a binary logistic regression model. We assessed whether more sophisticated methods offer advantages over this simple approach. It is often necessary to select among several candidate biomarker combinations. One strategy involves selecting a combination based on its ability to predict the outcome level of interest. We propose an algorithm that leverages the ordinal outcome to inform combination selection. We apply this algorithm to data from a study of acute kidney injury after cardiac surgery, where kidney injury may be absent, mild, or severe.

**Results:**

Using more sophisticated modeling approaches to construct combinations provided gains over the simple binary logistic regression approach in specific settings. In the examples considered, the proposed algorithm for combination selection tended to reduce the impact of bias due to selection and to provide combinations with improved performance.

**Conclusions:**

Methods that utilize the ordinal nature of the outcome in the construction and/or selection of biomarker combinations have the potential to yield better combinations.

**Electronic supplementary material:**

The online version of this article (10.1186/s41512-018-0028-3) contains supplementary material, which is available to authorized users.

## Background

In some clinical settings, a patient can experience one of several outcomes. For example, he can have no, mild, or severe disease. In the setting of cancer diagnosis, a patient can be disease-free or have cancer at one of several grades. However, it may be most important to be able to predict one particular level of the outcome, typically the level that poses the greatest health threat. In the examples just given, this may be severe disease or the presence of high-grade cancer. Here, investigators are interested in “single-level prediction,” but an ordinal outcome is available. Ordinal outcomes are polytomous, or multilevel, outcomes whose levels can be ordered by, for example, their clinical significance. In contrast, nominal outcomes are polytomous outcomes whose levels cannot be ordered. The question becomes whether and how the information from the ordinal outcome can be leveraged to improve prediction of the outcome level of interest.

It is becoming increasingly common for studies to measure several biomarkers in each participant. Such studies often seek to develop a combination of biomarkers that can be used in risk prediction. Developing a biomarker combination involves fitting, or constructing, a combination and, if more than one combination is available, selecting from among the candidates. When an ordinal outcome is available, the development of such combinations becomes potentially more complicated; we consider two such complications.

The first complication relates to the construction of biomarker combinations, specifically, how the biomarkers should be combined when an ordinal outcome is available but there is interest in single-level prediction. A natural and common approach is to dichotomize the outcome and fit a binary logistic regression model. Of course, this discards some information available in the ordinal outcome. We evaluate the potential benefits of alternative regression methods that utilize the ordinal outcome.

The second complication concerns how a biomarker combination should be selected. In many studies, the number of candidate biomarker combinations is quite large. Investigators may consider, for example, all possible pairs of biomarkers. In a study with 20 biomarkers, there would be nearly 200 such candidate combinations. One strategy is to choose the combination with the best performance in terms of single-level prediction. As with combination construction, it may be possible to leverage the additional information in the ordinal outcome to aid in combination selection. We propose an algorithm for doing so and provide examples to illustrate the benefits of this method.

We illustrate the application of our combination selection algorithm to data from the Translational Research Investigating Biomarker Endpoints in Acute Kidney Injury (TRIBE-AKI) study, a study of acute kidney injury (AKI) after cardiac surgery [1]. This study aims to use biomarkers measured immediately after surgery to provide an earlier diagnosis of AKI, which is typically not diagnosed until several days after surgery. Clinical definitions of AKI include both mild and severe types, though severe AKI is of primary clinical interest due to its impact on long-term morbidity and mortality [2]. As a result, there is interest in developing a biomarker combination to diagnose severe AKI.

### Constructing combinations

We consider a set of predictors **X** and an outcome *D* with *K* levels.

#### Models for binary outcomes

One set of regression-based approaches involves treating the outcome as binary by first dichotomizing *D* and/or subsetting the data and subsequently fitting one or more binary logistic regression models [3–7]. These approaches include:

(i) *Simple.* One binary logistic model based on dichotomizing *D* at some fixed level, *k*^′^: logit{*P*(*D*≤*k*^′^|**X**=**x**)}=*α*+***β***^*T*^**x** [8–12].
(ii) *Each level vs. others.K* binary logistic models comparing each level to the combination of the other levels:  [13, 14].
(iii) *Each level vs. reference.* (*K*−1) binary logistic models comparing each level to a reference level *k*^∗^:  [15, 16].
(iv) *Sequential.* (*K*−1) binary logistic models comparing each level to the combination of the levels above it:  etc. [13].

#### Models for nominal outcomes

The baseline-category logit model is a very flexible approach that considers the categorical nature of the outcome but does not incorporate the ordering [7, 17, 18]. The baseline-category logit model, typically referred to as the “multinomial model,” can be written as 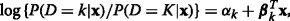 *k*=1,…,*K*−1 [17]. Thus, the baseline-category logit model allows the effect of the predictors to vary with the level of the outcome [17]. The set of models specified by the each level vs. reference approach (defined in the “Models for binary outcomes” section) with *k*^∗^=*K* as the reference level is parametrically equivalent to the baseline-category logit model.

#### Models for ordinal outcomes

Several regression models are available that fully model *D* (i.e., do not combine different levels of the outcome together) while accounting for the ordered, categorical nature of *D* [7, 17]. Such ordinal methods do not assume equal spacing between the levels of *D*; they simply use the ordering of the levels of *D* [19].

##### Cumulative logit model

The cumulative logit model can be written as 
1

*k*=1,…,*K*−1, where the *α*_*k*_ are ordered in *k* [17]. Under model (1), the log cumulative odds ratio is proportional to the distance between the predictor values being compared and the proportionality constant does not depend on *k* [17]: 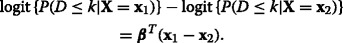


As a result of this proportionality (sometimes referred to as the parallel slopes assumption), model (1) is also called the proportional odds model [17]. It is possible to include a separate ***β*** vector, ***β***_*k*_, for each value of *k* [4, 17, 20, 21]. However, doing so may lead to crossing of the cumulative probability curves *P*(*D*≤*k*|**X**=**x**) for some values of **X**, violating the ordering of the cumulative probabilities [17, 20]. In other words, for a given value **x**, *P*(*D*≤1|**X**=**x**) may exceed *P*(*D*≤2|**X**=**x**), for example, which is not valid. Importantly, the estimates provided by the cumulative logit model may be biased under case-control sampling [12, 22].

##### Adjacent-category logit model

The adjacent-category logit model can be written as 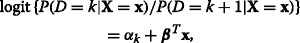 *k*=1,…,*K*−1 [17]. The adjacent-category logit model can be used with data from case-control studies [17]. The set of logits produced by the adjacent-category logit model is equivalent to that produced by the baseline-category logit model (defined in the “Models for nominal outcomes” section), except that the adjacent-category logit model assumes a common ***β*** [17]. Thus, the adjacent-category logit model takes advantage of the ordinal outcome to achieve parsimony but does not involve cumulative probabilities [17, 20]. The adjacent-category logit is more natural when there is interest in describing the effect of the predictor in terms of the odds relating to particular outcome levels since it easily allows the comparison of any two levels of the outcome [20]. Importantly, the issue discussed above for the cumulative logit model with separate effects ***β***_*k*_ (i.e., potential crossing of the cumulative probability curves, which violates the ordering of the cumulative probabilities) is not a problem for the adjacent-category logit model with separate effects since this model does not involve cumulative probabilities [17].

##### Continuation-ratio logit model

The continuation-ratio logit model can be written as 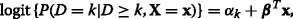 *k*=1,…,*K*−1, where the *α*_*k*_ are ordered in *k* [17]. The continuation-ratio logit model may be useful when a sequential mechanism determines the outcome, i.e., when individuals have to “pass through” one level of the outcome to get to the next [17, 19]. The continuation-ratio logit model considers conditional probabilities as opposed to cumulative probabilities [19]. As with the cumulative logit model, the continuation-ratio logit model restricts the regression coefficients to be the same for all levels of *k* [19, 21, 23]. Allowing a separate ***β*** vector for each levels of *k* gives the sequential approach defined in the “Models for binary outcomes” section [4, 19]. The estimates provided by the continuation-ratio logit model may be biased under case-control sampling [12].

##### Stereotype model

The stereotype model was proposed by Anderson [24] as a sort of compromise between models that incorporate the ordinality of the outcome and more flexible models (i.e., the baseline-category logit model defined in the “Models for nominal outcomes” section, which allows the entire coefficient vector to vary with *k*). The stereotype model actually includes a hierarchy of models that vary in flexibility, as defined by the dimension of the model [24]. The dimension of the model can range from one to the maximum dimension *d*, which is related to the number of predictors and outcome levels [24]. A stereotype model of maximum dimension is a reparameterization of the baseline-category logit model (defined in the “Models for nominal outcomes” section) [25]. While different dimensions are possible, the term “stereotype model” is generally reserved for the one-dimensional model and we focus on that model here. This model can be written as 
2

*k*=1,…,*K*−1 [24]. Essentially, this model allows some variation in the coefficient vector, but restricts ***β***_*k*_=*ϕ*_*k*_***β*** [24]. Identifiability constraints must be imposed on the *ϕ*_*k*_; typically, these are *ϕ*_1_=0 and *ϕ*_*K*_=1 [24]. The definition of the stereotype model also typically includes the requirement that *ϕ*_1_<*ϕ*_2_<…<*ϕ*_*K*_; when this holds, the model given in (2) is an ordered model [24]. However, it has been noted that this ordering does not need to be specified a priori and most statistical packages (e.g., R and Stata) do not impose such a restriction [4, 25]. In his examples, Anderson [24] did not assume ordering among the *ϕ*_*k*_; rather, he fit the one-dimensional model and evaluated whether the estimates $\hat {\phi }_{k}$ were ordered. Thus, Anderson [24] recommended fitting a fairly flexible model and assessing whether the data suggest ordering. In other words, the model allows users to judge whether the outcome levels are ordered or not based on the estimates $\hat {\phi }_{k}$, giving a data-driven analysis of ordering [23]. The stereotype model can be used with case-control data [12].

#### Comparing modeling approaches

Some work has been done to compare the models described above, particularly in terms of the efficiency of the parameter estimators. Briefly, gains in efficiency have been found for the baseline-category logit model relative to the each level vs. reference approach [15, 16], for the cumulative logit model relative to the simple approach [4, 6, 9], and for the stereotype model relative to the baseline-category logit model [26]. These results suggest that information can be gained from using all of the data in a single model, not dichotomizing *D*, and/or incorporating the ordinal nature of *D*.

Armstrong and Sloan concluded that in general, if the order of the outcome levels can be specified with confidence, models for ordinal outcomes, such as the cumulative logit model or the continuation-ratio logit model, are preferable to more flexible models [4]. In other words, it is reasonable to expect that when the outcome is ordinal, information is gained when this ordinality is used by the model [12]. In comparing the cumulative logit model to the simple approach, Risselada et al. argued that the impact of “mild violations” of the proportional odds assumption is expected to be less severe than the loss of information resulting from dichotomizing *D* [7]. On the other hand, others have noted that ordinal models become “increasingly unrealistic” as the number of outcome levels and/or predictors increases [9, 26].

#### Applications in risk prediction

Polytomous outcomes are frequently encountered in the risk prediction setting and a common approach is to dichotomize the outcome and fit a binary logistic regression model (the simple approach defined above) [3, 7, 14, 27]. The literature on using polytomous outcomes for single-level prediction has largely focused on the area under the receiver operating characteristic (ROC) curve (AUC) as a measure of predictive capacity. Briefly, the AUC assesses the ability of a model to discriminate between individuals who have or will experience the outcome level of interest and those who do not have or will not experience the outcome level of interest; the AUC for a model that is able to perfectly separate these groups is 1, while the AUC for a useless model is 0.5 [28].

The previous work in this area has primarily involved using individual datasets to compare modeling strategies. Biesheuvel et al. compared the baseline-category logit model to the sequential approach and found fairly similar AUCs for both strategies [14]. Roukema et al. compared the baseline-category logit model, the sequential approach, and the each level vs. others strategy [13]. They found similar discriminatory power for all three strategies, though they employed variable selection procedures for all of the models, making comparisons difficult [13]. Harrell et al. note that models can exhibit lack of fit and yet still provide quite accurate predicted probabilities [29], which may explain why several studies have found similar results in terms of the AUC when comparing different modeling approaches. In particular, it may be the case that a given model fits well for some predictors and does not fit as well for others, but when the coefficient estimates are combined to calculate predicted probabilities, the result is a fairly accurate estimate.

### Combination selection

Often, a number of candidate biomarker combinations are available, and some form of selection is required. When the goal is to use a biomarker combination for risk prediction, it seems appropriate to select combinations based on predictive capacity. For a binary outcome, one possibility is to use the AUC (e.g., [30]). That is, the AUC for each candidate combination is estimated, and the combination with the highest AUC is chosen.

Two challenges arise in utilizing this approach. The first is that when the same data are used to construct a biomarker combination and estimate the AUC (or other measure of performance) for that combination, the resulting AUC estimate will be optimistic relative to the AUC for the same fitted combination in independent data; we refer to this as “resubstitution bias” [31]. Methods such as bootstrapping can be used to correct the apparent AUC estimate [19].

An additional challenge applies to selection more generally. If many models are considered and a model is selected on the basis of some estimated measure of performance, that estimated measure of performance will be optimistically biased even if it is corrected for resubstitution bias; we refer to this as “model selection bias” [31]. This idea has been explored in the bioinformatics/machine learning literature, where estimates of the classification error rate are often used to select a model. Broadly, it has been found that the estimated error rate for a model selected on the basis of its favorable error rate will be optimistic relative to the same model’s error rate in independent data [32–38]. Cawley and Talbot call this issue “overfitting the model selection criterion” [34]. In general, when some form of model selection is done and the performance of the chosen model is evaluated without accounting for the selection, that is, treating the selected model as though it were pre-specified, optimistic bias is expected [19, 35, 39–41].

## Methods

For an outcome *D* with *K* levels, “single-level prediction” relates to predicting *D*=*K*. In other words, we are interested in developing a biomarker combination that can differentiate *D*=*K* from *D*<*K*. Thus, we focus on measures of performance that evaluate the model in this regard. However, this begs the question of whether the ordered, multilevel nature of the outcome can be used in constructing and/or selecting a biomarker combination.

### Methods for constructing combinations

We have described several regression-based approaches to modeling polytomous outcomes. In particular, we can dichotomize the outcome and/or subset the data and use one of the four binary strategies, we can treat the outcome as ordered and use one of the four ordinal approaches, or we can use the more flexible baseline-category logit model. Using a binary strategy requires combining several levels of the outcome together or fitting several models to subsets of the data. Likewise, the ordinal models restrict the nature of the relationship between the biomarkers and the outcome so as to achieve parsimony. The baseline-category logit model, on the other hand, imposes no such restrictions and includes all of the data in a single model; of course, this comes at the cost of having to estimate additional parameters. We use simulations to evaluate the impact of these modeling choices on the performance of the resulting estimated combinations. Our focus in this investigation is whether more sophisticated modeling approaches can offer improvements in performance in terms of single-level prediction over the simple approach, that is, a single binary logistic regression model.

Since we are interested in predicting *D*=*K*, we dichotomized *D* at *k*^′^=*K*−1 in the simple approach defined in the “Models for binary outcomes” section, giving logit{*P*(*D*≤*K*−1)}. Furthermore, for the purposes of predicting *D*=*K*, the simple approach and the each level vs. others strategy are identical, so the latter was not considered further. Finally, as the baseline-category logit model is parametrically equivalent to the each level vs. reference approach defined in the “Models for binary outcomes” section with reference level *k*^∗^=*K* and the former is generally more efficient than the latter, we did not include the each level vs. reference strategy in our investigation. Thus, we considered seven different modeling strategies: the simple approach (“Simple”), the sequential strategy (“Sequential”), the cumulative logit model (“CumLogit”), the adjacent-category logit model (“AdjCatLogit”), the continuation-ratio logit model (“ContRatLogit”), the stereotype model (“Stereo”), and the baseline-category logit model (“BaselineCat”).

We considered two broad simulation scenarios. In the first scenario, the biomarkers were simulated such that the cumulative logit model with proportional odds did not hold; in the second scenario, the data were simulated under the cumulative logit model where the assumption of proportional odds held. In both scenarios, we considered two biomarkers, **X**=(*X*_1_,*X*_2_). We considered outcomes with either 3 or 5 levels, that is, *K*=3 or *K*=5. The combinations were constructed using training data with 200, 400, 800, or 1600 observations and evaluated in test data with 10^4^ observations. The training set sizes reflect sample sizes often encountered in biomarker studies. We used large test sets to provide reliable estimates of model performance. We simulated data such that *P*(*D*=1)=0.1 or 0.5 and *P*(*D*=*K*)=0.05 or 0.3; when *K*=5, *P*(*D*=2), *P*(*D*=3), and *P*(*D*=4) were equal. Thus, we considered scenarios where the outcome level *K*, the target of prediction, was rare and scenarios where it was common.

We used each of the modeling strategies to fit a linear combination of the biomarkers **X** in the training data, yielding estimates $\hat {\boldsymbol {\beta }}$. We then applied these estimates to the test data to determine . Finally, we assessed the ability of  to discriminate between *D*=*K* and *D*<*K* in the test data via the AUC. In the AKI example given above, this is the AUC for severe AKI vs. no or mild AKI. As we are interested in single-level prediction, this measure is the most relevant metric by which to compare the methods.

In the simulations where the cumulative logit model with proportional odds did not hold (the first scenario mentioned above), the biomarkers had conditional bivariate normal distributions. In particular, for *K* = 3, we considered (**X**|*D* = 1)∼*N*(**0**,*Σ*), (**X**|*D* = 2) ∼ *N*(***μ***,*Σ*), and (**X**|*D* = 3)∼*N*(**2**,*Σ*), and for *K* = 5, we considered (**X**|*D* = 1)∼*N*(**0**,*Σ*), (**X**|*D* = 2)∼*N*(**0.5**,*Σ*), (**X**|*D* = 3)∼*N*(**1**,*Σ*), (**X**|*D* = 4)∼*N*(***μ***,*Σ*), and (**X**|*D* = 5)∼*N*(**2**,*Σ*). We used ***μ***∈{−**1**,**0**,**1**,**2**,**3**} and *Σ* = 2*I*_2_, where *I*_2_ is the two-dimensional identity matrix. Other covariance matrices (including those with correlation between the biomarkers and unequal covariance matrices) were explored; details are given in (Additional file 1: Section S1.1). The parameter ***μ*** determines whether the biomarker means are ordered by *D*. For *K* = 3, when ***μ*** is between **0** and **2**, the biomarker means can be ordered by *D*. The same is true for *K* = 5 when ***μ*** is between **1** and **2**. When the biomarker means were not ordered by *D*, we anticipated that some of the ordinal and/or binary methods would not perform well; these scenarios were included to investigate situations where the expected ordering of an outcome is not borne out by the data. We considered conditional bivariate normal distributions in these simulations because they yield the baseline-category logit model.

To evaluate data generated by the cumulative logit model with proportional odds (second scenario), we simulated two independent normal biomarkers, both with mean 1 and variance 0.25. The outcome was then simulated as a multinomial random variable, where the success probabilities of the *K* levels were determined by *α*_*k*_−***β***^⊤^**X** such that the cumulative logit model held. Three sets of coefficients ***β*** were considered (***β***=(1,2),(1,1.5),(1,−1)) and values of *α*_*k*_ were chosen such that the desired prevalences (given above) were achieved in a large dataset. We chose to use normally distributed biomarkers in this simulation as, in our experience, many biomarkers are approximately normally distributed after a log transformation.

The simulations were repeated 1000 times and are described in Table 1.

**Table 1:** Description of simulation scenarios for combination construction

Data-generating model	*K*	Training sample size	Prevalences	Biomarker distributions	Parameters
Non-proportional odds	3	200, 400, 800, 1600	*P*(*D*=1)=0.1,0.5	(**X**|*D*=1)∼*N*(**0**,2*I*_2_)	***μ***∈{**–1**,**0**,**1**,**2**,**3**}
			*P*(*D*=*K*)=0.05,0.3	(**X**|*D*=2)∼*N*(***μ***,2*I*_2_)	
				(**X**|*D*=3)∼*N*(**2**,2*I*_2_)	
	5	200, 400, 800, 1600	*P*(*D*=1)=0.1,0.5	(**X**|*D*=1)∼*N*(**0**,2*I*_2_)	***μ***∈{**–1**,**0**,**1**,**2**,**3**}
			*P*(*D*=*K*)=0.05,0.3	(**X**|*D*=2)∼*N*(**0.5**,2*I*_2_)	
				(**X**|*D*=3)∼*N*(**1**,2*I*_2_)	
				(**X**|*D*=4)∼*N*(***μ***,2*I*_2_)	
				(**X**|*D*=5)∼*N*(**2**,2*I*_2_)	
Proportional odds	3	200, 400, 800, 1600	*P*(*D*=1)=0.1,0.5	*X*_1_∼*N*(1,0.25)	(*β*_1_,*β*_2_)∈{(1,2),
			*P*(*D*=*K*)=0.05,0.3	*X*_2_∼*N*(1,0.25)	(1,1.5),(−1,1)}
	5	200, 400, 800, 1600	*P*(*D*=1)=0.1,0.5	*X*_1_∼*N*(1,0.25)	(*β*_1_,*β*_2_)∈{(1,2),
			*P*(*D*=*K*)=0.05,0.3	*X*_2_∼*N*(1,0.25)	(1,1.5),(−1,1)}

When *K*=5, *P*(*D*=2)=*P*(*D*=3)=*P*(*D*=4). For the proportional odds data-generating model, logit{*P*(*D*≤*k*|*X*_1_,*X*_2_)}=*α*_*k*_+*β*_1_*X*_1_+*β*_2_*X*_2_. *I*_2_ is the two-dimensional identity matrix

### Methods for combination selection

As above, we suppose that for an outcome *D* with *K* levels, “single-level prediction” relates to predicting *D*=*K*.

As with combination construction, the presence of an ordinal outcome requires that decisions about how to select a biomarker combination be made. One strategy is to simply estimate the AUC for *D*=*K* vs. *D*<*K* (including correcting this estimate for resubstitution bias due to any model fitting) and select the combination with the highest estimated AUC. As discussed above, the estimated AUC for this selected combination will be optimistically biased relative to the AUC for the same fitted combination in independent data due to model selection bias. In other words, because of model selection bias, the estimated AUC for the selected combination, chosen precisely because of its high estimated AUC, will be optimistically biased compared to the performance of this fitted combination in external data. We propose an alternative strategy where combination selection is done on the basis of not only the AUC for *D*=*K* vs. *D*<*K*, but also the AUC for *D*=*K*−1 vs. *D*<*K*−1, the AUC for *D*=*K*−2 vs. *D*<*K*−2, and so on.

We anticipate that in some settings, the estimated AUC for *D*=*K* vs. *D*<*K* for the combination selected in this way will be less affected by model selection bias and so this combination may be preferred. In particular, if some of the same biomarkers are associated with multiple levels of the outcome, our proposed method could offer improvements over the standard approach. Furthermore, we expect our approach to be useful when many biomarkers have modest associations with the outcome and the candidate combinations include subsets of these biomarkers. For example, if two biomarkers have very strong associations with *D*=*K* vs. *D*<*K* (i.e., the biomarkers for *D*=*K* are very different from those for *D*<*K*) and the remainder have much weaker associations, if we consider all biomarker pairs, the AUC for *D*=*K* vs. *D*<*K* for the pair consisting of the two strongly associated biomarkers is expected to be much larger than this AUC for the other combinations and it is unlikely this difference is due entirely to model selection bias. Thus, in this scenario, the “standard” approach of selecting a combination on the basis of the estimated AUC for *D*=*K* vs. *D*<*K* would be expected to yield the best combination in terms of the true AUC for *D*=*K* vs. *D*<*K*.

More precisely, for *K*=3, we define our algorithm (including constructing combinations and estimating their performance) as follows.

1. In the training data, dichotomize *D* at *D*=3 vs. *D*<3 and construct all candidate biomarker combinations using binary logistic regression. That is, to construct a candidate combination involving biomarkers **X**, fit logit{*P*(*D*=3|**X**=**x**)}=*θ*_0_+***θ***^⊤^**x** in the training data.
2. Based on the fitted combinations from (1), e.g., $\hat {\theta }_{0} + \hat {\boldsymbol {\theta }}^{\top }\mathbf {x}$, estimate (i) the AUC for *D*=3 vs. *D*<3 and (ii) the AUC for *D*=2 vs. *D*=1 in the training data.
3. Generate *B* bootstrap samples from the training data.
  - In each bootstrap sample, dichotomize *D* at *D*=3 vs. *D*<3 and construct all candidate biomarker combinations using binary logistic regression.
  - For each of the fitted combinations from (a), estimate (i) the AUC for *D*=3 vs. *D*<3 and (ii) the AUC for *D*=2 vs. *D*=1 in both the bootstrap sample and the training data.
  - Estimate the resubstitution bias as the average difference between the AUC in the bootstrap sample and the AUC in the training data across the *B* samples.
4. Correct the estimated AUCs from (2) using the estimated bias from (3c).
5. Determine the ranks for each of the two sets of corrected AUCs from (4) across all fitted biomarker combinations. The “standard” approach involves choosing the combination with the best AUC for *D*=3 vs. *D*<3. The “new” approach involves choosing the combination with the best sum of ranks for the two AUCs.
6. Apply the two chosen combinations to independent test data and estimate the AUC for *D*=3 vs. *D*<3 for each of the two selected combinations from (5). The estimated model selection bias is the difference between the AUCs in the test data and the AUCs from (4).

In practice, test data may not be available, so it may not be possible to complete step (6). An R package including code to implement this method, multiselect, is available on CRAN.

We used simulations to investigate the potential benefits of the proposed method. We considered five examples as a proof of concept; these are not intended to be exhaustive. In the first two examples, the cumulative logit model with proportional odds held, while in the other three, it did not. Throughout the simulations, *K*=3, there were *p*=30 biomarkers, and we considered the set of candidate combinations to be all possible pairs of these biomarkers, constructed via binary logistic regression. We used *B*=50 bootstrap replicates, a training set of 400 observations, and a test set of 10^4^ observations. We repeated the simulations 500 times. Our choice of *p* represents a study with a modest, but not large, number of biomarkers. As with the combination construction simulations, we chose a training set size similar to what might be encountered in a cohort study and a test set size that would yield reliable AUC estimates. We chose *B*=50 to provide computational efficiency without sacrificing performance. In these simulations, we considered all biomarker pairs to be candidate combinations. However, the number of biomarkers to be selected may not be fixed in practice. Our method is expected to would work equally well if the set of candidate combinations was all combinations of, say, between two and five biomarkers.

In Example 1, we had **X**∼*N*(**1**,2*Γ*), where **X** was a vector of dimension 30 and *Γ* was a 30×30 matrix where the diagonal elements were 1 and the off-diagonal elements were 0.3. The linear predictor was ***β***^⊤^**X**, where *β*_1_ = 1,*β*_2_=2,*β*_3_=…=*β*_16_=0.5,*β*_17_=…=*β*_30_=0.1. The outcome was simulated under the cumulative logit model such that *P*(*D*=1)=0.6, *P*(*D*=2)=0.3, and *P*(*D*=3)=0.1 in a large dataset. Example 2 was identical to Example 1, except that *P*(*D*=2)=0.335 and *P*(*D*=3)=0.065.

In Example 3, we had *P*(*D*=1)=0.6, *P*(*D*=2)=0.335, and *P*(*D*=3)=0.065. Additionally, (**X**|*D*=1)∼*N*(**0**,2*Γ*),(**X**|*D*=2)∼*N*(***β***^(2)^,2*Γ*), and (**X**|*D*=3)∼*N*(***β***^(3)^,2*Γ*) where **X** was a vector of dimension 30, *Γ* was as defined above for Example 1, and $\beta ^{(2)}_{1} = 1.5, \beta ^{(2)}_{2} = 1, \beta ^{(2)}_{3} = \ldots = \beta ^{(2)}_{16} = 0.5, \beta ^{(2)}_{17} = \ldots = \beta ^{(2)}_{30} = 0.1, \beta ^{(3)}_{1} = \beta ^{(3)}_{2} = 2, \beta ^{(3)}_{3} = \ldots = \beta ^{(3)}_{16} = 0.8$, and $\beta ^{(3)}_{17} = \ldots = \beta ^{(3)}_{30} = 0.1.$ Example 4 was identical to Example 3, except that $\beta ^{(2)}_{1} = 1$ and $\beta ^{(3)}_{17} = \ldots = \beta ^{(3)}_{30} = 0.2.$ Finally, Example 5 was identical to Example 3, except that $\beta ^{(2)}_{1} = 1$, $\beta ^{(2)}_{17} = \ldots = \beta ^{(2)}_{30} = 0$, and $\beta ^{(3)}_{17} = \ldots = \beta ^{(3)}_{30} = 0.2$.

These examples reflect a range of scenarios, including situations where *D*=3 is common and situations where it is relatively rare. The biomarkers in these examples are moderately correlated, as might be expected in practice. We considered scenarios where two biomarkers had stronger effects on *D* and the remaining biomarkers either had more modest effects or were not associated with some levels of *D*. In Examples 1 and 2, “stronger effects” mean *β* values farther from zero, which translate into odds ratios (where the odds correspond to *P*(*D*≤*k*)/ *P*(*D*>*k*)) that are farther from one. For Examples 3–5, “stronger effects” mean *β*^(*k*)^ farther from zero, which translate into biomarker means for *D*=*k* that are more different from those for *D*=1.

## Results

### Results for constructing combinations

First we consider the scenario where the cumulative logit model with proportional odds did not hold. We present the results for a training set size of 400; the results for the other sample sizes were similar. Here, we focus on the results for *P*(*D*=*K*)=0.05 and provide the full results in (Additional file 1: Section S1.1).

Table 2 presents the results for *K*=3 and Table 3 presents the results for *K*=5. We see that when ***μ***=−**1**, ***μ***=**0**, or ***μ***=**1**, the simple approach was comparable to or better than the other approaches. When ***μ***=**2**, the simple approach did slightly worse than some of the ordinal approaches, particularly for *K*=3. For ***μ***=**3**, the sequential approach, the stereotype model, and/or the baseline-category logit model offered some gains over the simple approach. In sum, when the cumulative logit model with proportional odds did not hold but there was some ordering in the outcome by the biomarkers (that is, ***μ***<2), the simple approach did well, but when ***μ***≥2, some of the alternative approaches demonstrated improved performance. This is expected, since when ***μ***<2, the simple approach is able to separate *D*<*K* from *D*=*K*, and so performs well. Furthermore, as indicated above, it is not surprising that when ***μ*** was extreme, many of the ordinal and binary approaches did not perform well.

**Table 2:** Simulation results for *K*=3

Class	Model	*μ*=−1	*μ*=0	*μ*=1	*μ*=2	*μ*=3
P(D=1) = 0.1						
Binary	Simple	0.976 (0.974, 0.978)	0.920 (0.915, 0.924)	0.773 (0.764, 0.780)	0.530 (0.508, 0.543)	0.670 (0.642, 0.684)
	Sequential	0.974 (0.971, 0.976)	0.920 (0.915, 0.924)	0.773 (0.764, 0.780)	0.532 (0.519, 0.544)	0.720 (0.708, 0.729)
Nominal	BaselineCat	0.976 (0.974, 0.978)	0.920 (0.915, 0.924)	0.773 (0.764, 0.780)	0.532 (0.519, 0.544)	0.720 (0.707, 0.728)
Ordinal	CumLogit	0.970 (0.946, 0.975)	0.918 (0.912, 0.923)	0.776 (0.769, 0.783)	0.544 (0.536, 0.552)	0.313 (0.306, 0.320)
	AdjCatLogit	0.970 (0.952, 0.975)	0.918 (0.912, 0.923)	0.776 (0.769, 0.783)	0.544 (0.536, 0.552)	0.313 (0.306, 0.320)
	ContRatLogit	0.971 (0.958, 0.976)	0.918 (0.912, 0.923)	0.776 (0.769, 0.783)	0.544 (0.536, 0.552)	0.313 (0.306, 0.320)
	Stereo	0.976 (0.974, 0.978)	0.920 (0.915, 0.924)	0.776 (0.769, 0.783)	0.535 (0.520, 0.547)	0.724 (0.715, 0.732)
P(D=1) = 0.5						
Binary	Simple	0.950 (0.946, 0.952)	0.920 (0.915, 0.924)	0.841 (0.834, 0.848)	0.714 (0.705, 0.723)	0.588 (0.571, 0.599)
	Sequential	0.924 (0.911, 0.933)	0.919 (0.915, 0.924)	0.842 (0.834, 0.848)	0.712 (0.701, 0.722)	0.743 (0.733, 0.752)
Nominal	BaselineCat	0.950 (0.946, 0.952)	0.920 (0.915, 0.924)	0.841 (0.835, 0.848)	0.712 (0.702, 0.722)	0.743 (0.733, 0.752)
Ordinal	CumLogit	0.054 (0.050, 0.062)	0.916 (0.907, 0.921)	0.844 (0.838, 0.849)	0.721 (0.715, 0.728)	0.599 (0.593, 0.604)
	AdjCatLogit	0.073 (0.054, 0.198)	0.917 (0.911, 0.922)	0.844 (0.838, 0.849)	0.721 (0.715, 0.728)	0.599 (0.593, 0.604)
	ContRatLogit	0.094 (0.057, 0.409)	0.917 (0.911, 0.922)	0.844 (0.838, 0.849)	0.721 (0.715, 0.728)	0.599 (0.593, 0.604)
	Stereo	0.950 (0.947, 0.953)	0.920 (0.915, 0.924)	0.844 (0.838, 0.849)	0.718 (0.709, 0.725)	0.749 (0.741, 0.756)

Results for *n*=400 and *P*(*D*=3)=0.05 when the cumulative logit model with proportional odds did not hold. The table presents the median and interquartile range of the AUCs for *D*=*K* vs. *D*<*K* in the test data for the combinations fitted by each modeling strategy

**Table 3:** Simulation results for *K*=5

Class	Model	*μ*=−1	*μ*=0	*μ*=1	*μ*=2	*μ*=3
P(D=1) = 0.1						
Binary	Simple	0.870 (0.864, 0.875)	0.851 (0.844, 0.856)	0.802 (0.794, 0.810)	0.721 (0.710, 0.730)	0.636 (0.615, 0.647)
	Sequential	0.732 (0.699, 0.760)	0.836 (0.824, 0.845)	0.802 (0.793, 0.810)	0.720 (0.708, 0.729)	0.693 (0.681, 0.703)
Nominal	BaselineCat	0.870 (0.864, 0.875)	0.851 (0.844, 0.857)	0.802 (0.794, 0.810)	0.720 (0.709, 0.729)	0.696 (0.684, 0.704)
Ordinal	CumLogit	0.134 (0.128, 0.144)	0.804 (0.673, 0.843)	0.804 (0.797, 0.811)	0.728 (0.721, 0.735)	0.650 (0.643, 0.656)
	AdjCatLogit	0.140 (0.130, 0.161)	0.831 (0.781, 0.847)	0.804 (0.797, 0.811)	0.728 (0.721, 0.735)	0.650 (0.643, 0.656)
	ContRatLogit	0.138 (0.130, 0.158)	0.810 (0.690, 0.844)	0.804 (0.796, 0.811)	0.728 (0.721, 0.735)	0.650 (0.643, 0.656)
	Stereo	0.872 (0.867, 0.877)	0.853 (0.847, 0.858)	0.804 (0.797, 0.811)	0.727 (0.718, 0.734)	0.701 (0.692, 0.709)
P(D=1) = 0.5						
Binary	Simple	0.893 (0.888, 0.898)	0.883 (0.877, 0.888)	0.856 (0.850, 0.862)	0.814 (0.807, 0.821)	0.769 (0.757, 0.777)
	Sequential	0.791 (0.756, 0.824)	0.878 (0.869, 0.884)	0.856 (0.850, 0.862)	0.814 (0.807, 0.820)	0.790 (0.780, 0.798)
Nominal	BaselineCat	0.893 (0.888, 0.898)	0.883 (0.877, 0.888)	0.857 (0.851, 0.863)	0.815 (0.808, 0.821)	0.793 (0.786, 0.800)
Ordinal	CumLogit	0.878 (0.828, 0.891)	0.883 (0.877, 0.888)	0.858 (0.853, 0.864)	0.818 (0.813, 0.824)	0.777 (0.772, 0.782)
	AdjCatLogit	0.866 (0.773, 0.889)	0.883 (0.876, 0.888)	0.858 (0.853, 0.864)	0.819 (0.813, 0.824)	0.777 (0.772, 0.782)
	ContRatLogit	0.852 (0.720, 0.886)	0.882 (0.875, 0.887)	0.858 (0.853, 0.864)	0.818 (0.813, 0.824)	0.777 (0.772, 0.782)
	Stereo	0.895 (0.890, 0.899)	0.884 (0.879, 0.889)	0.859 (0.853, 0.864)	0.818 (0.812, 0.824)	0.798 (0.790, 0.804)

Results for *n*=400 and *P*(*D*=5)=0.05 when the cumulative logit model with proportional odds did not hold. The table presents the median and interquartile range of the AUCs for *D*=*K* vs. *D*<*K* in the test data for the combinations fitted by each modeling strategy

While our main goal was to see if another regression method could yield improvements over the simple approach, it is instructive to note some of the patterns for the other methods. For instance, when *K*=3 and ***μ***=**3**, the cumulative logit, adjacent-category logit, and continuation-ratio logit models performed poorly relative to some of the other methods. This is not surprising as these methods are most appropriate when there is some ordering of the outcome, but when ***μ***=**3**, the mean of the biomarkers for *D*=2 exceeds the mean of the biomarkers for *D*=3. This had more of an impact for *K*=3 than for *K*=5, since for *K*=5, four out of the five outcome levels were still ordered and so there seems to have been less of an effect on the models’ performance.

Similarly, when *K*=3, *P*(*D*=1)=0.5, and ***μ***=**–1**, the cumulative logit, adjacent-category logit, and continuation-ratio logit models again did poorly compared to some of the alternatives. However, we did not see this when *P*(*D*=1)=0.1. This is because when *P*(*D*=1)=0.5, *P*(*D*=2)=0.45, but when *P*(*D*=1)=0.1, *P*(*D*=2)=0.85. Thus, when *P*(*D*=1)=0.1, the *D*=2 level essentially overwhelmed *D*=1, so the fact that *D*=2 is “out of order” had less of an impact on model fitting. On the other hand, when *P*(*D*=1)=0.5 and *P*(*D*=2)=0.45, both *D*=1 and *D*=2, and their lack of ordering relative to *D*=3, influenced model fitting. Likewise, when *K*=5, *P*(*D*=1)=0.1, and ***μ***=**–1**, the cumulative logit, adjacent-category logit, and continuation-ratio logit models did not perform well. A similar argument applies here, but in reverse: when *P*(*D*=1)=0.1, *P*(*D*=2)=*P*(*D*=3)=*P*(*D*=4)≈0.28, and when *P*(*D*=1)=0.5, *P*(*D*=2)=*P*(*D*=3)=*P*(*D*=4)=0.15. Thus, when *P*(*D*=1)=0.5, the “out of order” level, *D*=4, was overwhelmed by the other (ordered) levels, but when *P*(*D*=1)=0.1, *P*(*D*=4) is considerably higher, and the fact that *D*=4 is “out of order” had a larger influence on model fitting.

Finally, we note that in these situations, where the cumulative logit, adjacent-category logit, and continuation-ratio logit models performed poorly, the other ordinal approach, the stereotype model, offered enough flexibility to provide models with good performance.

When the cumulative logit model with proportional odds held, the performance was comparable across the approaches considered for a training set size of 400 (Additional file 1: Section S1.2); similar patterns were seen for other sample sizes. Thus, even when the data were generated by an ordinal model, the simple approach did well in terms of the predictive capacity of the fitted combinations.

For small to moderate sample sizes, several of the approaches had issues with convergence. When the training set had 200 observations, the simple approach failed to converge in up to 3.1% of simulations, the sequential approach failed to converge in up to 38% of simulations, the stereotype model failed to converge in up to 2.6% of simulations, and the baseline-category logit model failed to converge in up to 1.4% of simulations. For training data with 400 observations, the sequential approach failed to converge in up to 7% of simulations. The proportion of convergence failures was below 0.2% for all methods for larger sample sizes.

### Results for combination selection

Table 4 presents the results for Examples 1 and 4 for the proposed combination selection method. The results for Examples 2, 3, and 5 show similar patterns; the full results are presented in (Additional file 1: Section S2). The results in Table 4 demonstrate some benefit to using the additional information available in the ordinal outcome to select a biomarker combination for single-level prediction, both in terms of the degree of model selection bias and the ability of the chosen combination to discriminate *D*=3 from *D*<3 in independent test data.

**Table 4:** Results for the proposed combination selection method for Examples 1 and 4

	Method	Bias	AUC
Example 1	Standard	0.030 (0.020, 0.044)	0.911 (0.905, 0.917)
	New	0.014 (0.005, 0.026)	0.916 (0.911, 0.923)
Example 4	Standard	0.042 (0.012, 0.068)	0.794 (0.777, 0.834)
	New	0.010 (-0.018, 0.037)	0.831 (0.822, 0.838)

The table gives the median (interquartile range) of the estimated model selection bias and the AUC for *D*=3 vs. *D*<3 in test data for the combinations selected by the two approaches

In all of the examples considered, the first two biomarkers (*X*_1_ and *X*_2_) had larger coefficients than the other biomarkers. This suggests that the pair (*X*_1_,*X*_2_) may offer better performance than the other candidate biomarker pairs. To explore this, we investigated how often this pair was chosen by each method. In Example 1, the pair (*X*_1_,*X*_2_) was chosen in 19.2% of simulations by the standard approach and in 41.4% of simulations by the new approach. For Example 2, these numbers were 13.4 and 34.8%, respectively. In Example 3, they were 27.4 and 57.6%, and for Example 4, they were 44 and 82.4%. Finally, for Example 5, they were 50.2 and 79.8%.

There were no issues with the logistic regression model failing to converge in the Example 1 simulations, eight simulations (out of 500) had convergence issues in Example 2, and one simulation had convergence issues in each of Examples 3, 4, and 5.

#### Application to TRIBE-AKI

We applied our proposed method for combination selection to data from the TRIBE-AKI study. As noted above, the outcome in this study, AKI, is an ordinal outcome as patients may be diagnosed with no, mild, or severe AKI. Furthermore, of the biomarkers measured in the study, it is believed that only a subset are likely to be useful for early diagnosis. Thus, we considered all possible pairs of 14 biomarkers measured in the study.

In the TRIBE-AKI study, severe AKI is defined as a doubling of serum creatinine over preoperative levels or the need for dialysis during the hospital stay and mild AKI is defined as an increase in serum creatinine of 50%. The TRIBE-AKI study is a multicenter study, but we restricted attention to the largest center in order to avoid issues related to center differences. We used the biomarker measurements taken immediately after surgery and removed observations missing any of these measurements. This left 465 observations (61 with mild AKI and 30 with severe AKI). We also log-transformed the biomarker measurements. As in the simulations, we applied our proposed method with 50 bootstrap replications.

The results for the ten best combinations in terms of the AUC for severe vs. no/mild AKI are given in Table 5. The combination with the highest AUC for severe vs. no/mild AKI, which would be selected by the standard approach, includes urine interleukin-18 (IL-18) and plasma N-terminal-pro-B-type natriuretic peptide (NT-proBNP). The estimated AUCs (corrected for resubstitution bias) for this combination were 0.8575 for severe vs. no/mild AKI and 0.6125 for mild vs. no AKI. The combination with the highest combined rank for the AUC for severe vs. no/mild AKI and the AUC for mild vs. no AKI, which would be selected by the proposed method, included plasma heart-type fatty acid binding protein (h-FABP) and plasma interleukin-6 (IL-6). The estimated AUCs (corrected for resubstitution bias) for this combination were 0.8365 for severe vs. no/mild AKI and 0.6757 for mild vs. no AKI. Thus, the AUC for severe vs. no/mild AKI for this second combination is slightly lower, but the AUC for mild vs. no AKI is substantially higher. It may be reasonable to expect that the estimated AUC for severe vs. no/mild AKI for the second combination (0.8365) is less affected by model selection bias than is the estimated AUC for severe vs. no/mild AKI for the first combination (0.8575), which may motivate choosing to validate the second combination instead of the first.

**Table 5:** The ten best biomarker pairs in the TRIBE-AKI study

Biomarkers		AUC (Severe)	AUC (Mild)
Urine IL-18	Plasma NT-proBNP	0.8575	0.6125
Plasma h-FABP	Urine IL-18	0.8495	0.6394
Plasma h-FABP	Plasma BNP	0.8464	0.6403
Plasma h-FABP	Plasma NT-proBNP	0.8459	0.6329
Urine IL-18	Plasma BNP	0.8414	0.6168
Plasma h-FABP	Urine KIM-1	0.8410	0.6400
Plasma h-FABP	Plasma IL-6	0.8365	0.6757
Plasma h-FABP	Plasma IL-10	0.8342	0.6405
Plasma h-FABP	Plasma CKMB	0.8271	0.6558
Urine KIM-1	Plasma TNTHS	0.8253	0.6005

The table presents the ten pairs with the highest estimated AUC for severe vs. no/mild AKI. The estimated AUCs for severe vs. no/mild AKI and for mild vs. no AKI are presented. Both estimates are corrected for optimism due to resubstitution bias

## Discussion

When there is interest in developing biomarker combinations for single-level prediction of an ordinal outcome, common practice is to dichotomize the outcome for combination construction and selection. We have considered whether the information in an ordinal outcome can be leveraged in the development of biomarker combinations for single-level prediction.

In the context of constructing biomarker combinations, we used simulations to compare seven regression-based approaches: two binary approaches, four ordinal approaches, and one nominal approach. We considered a variety of data-generating scenarios and found that when some separation in the biomarker distributions between *D*=*K* and *D*<*K* existed (i.e., ***μ***<**2** in our first simulation scenario) or when the cumulative logit model with proportional odds held, the simple approach based on dichotomizing the outcome tended to work well in terms of the ability of the resulting combinations to predict *D*=*K*.

We have also proposed a method that utilizes the ordinal nature of the outcome in selecting a biomarker combination, as opposed to selecting a combination based solely on its ability to predict the targeted level. Simulations provide evidence that use of the proposed method may result in less model selection bias and could lead to selecting combinations with greater predictive capacity. We applied this method to data from the TRIBE-AKI study, where we demonstrated how the method could be used to select a combination in practice. This approach is expected to be most useful when there is some ordering in the biomarkers by the levels of *D*. It is important to study this method further in order to fully elucidate the settings in which it could be beneficial.

In using this method for selection, it is generally informative to look at the results for the candidate combinations, as we have done in Table 5 for the top ten pairs in the TRIBE-AKI study. If there is a clear “winner” in terms of the AUC for *D*=3 vs. *D*<3, that is, if this AUC is substantially higher for one candidate combination, it is probably reasonable to select that combination, regardless of the AUC for *D*=2 vs. *D*=1. This is because it is unlikely that such a markedly higher AUC estimate is due to model selection bias. On the other hand, if several combinations have fairly similar performance in terms of the AUC for *D*=3 vs. *D*<3, it may be worth using the AUC for *D*=2 vs. *D*=1 to aid in selection. One possible extension of this method could involve using a weighted average of ranks for the two AUCs, rather than the sum; additionally, using a weighted average of the AUC values themselves (as opposed to their ranks) could be considered.

The proposed method for selection is most appropriate when a modest number of biomarkers is available. When the number of candidate biomarkers is very large, some form of pre-selection may be required, for example, using p-values or some other measure of the univariate association between a biomarker and the outcome. However, univariate measures of association may not reflect a biomarker’s potential to improve prediction when combined with other predictors [42], so pre-selection is best avoided when feasible [43].

In some settings, a polytomous outcome arises from the categorization of a continuous outcome; in such instances, methods for continuous data may yield better performance. Our work is primarily concerned with situations where the outcome is not based on the categorization of a continuous variable, such as the AKI and cancer grade examples.

Several measures of performance for polytomous outcomes have been proposed, including the polytomous discrimination index [44], the ordinal *c*-index [45], and the hypervolume under the manifold [46], each of which generalizes the AUC from binary to polytomous outcomes. These measures summarize the ability of a combination to predict all levels of the outcome, and so are not well-suited to the work discussed here, which pertains to the situation where predicting a particular level of the outcome is the primary goal. Given this goal, we examined the AUC for *D*=*K* vs. *D*<*K* in constructing combinations. Additionally, while our selection algorithm uses information from multiple AUCs, we also recommend considering the magnitude of the AUC for *D*=*K* vs. *D*<*K* in selecting a combination. As noted above, a combination with a much higher AUC for *D*=*K* vs. *D*<*K* (relative to the other candidate combinations) should be preferred regardless of its ability to predict the other levels. Finally, we note that we have focused on the AUC as a measure of performance and a tool for combination selection. Alternative measures of discrimination may also be useful and could yield different results.

## Conclusions

When an ordinal outcome is available and there is interest in using biomarker combinations to predict a single level of the outcome, the common approach of dichotomizing the outcome to construct and/or select a combination necessarily discards some information. We have considered the utility of leveraging this information to advance the goal of single-level prediction.

## Additional file

Additional file 1 An additional file (“Additional File 1.pdf”) contains Sections S1 and S2. Section S1 contains results for simulations comparing methods for constructing combinations when the cumulative logit model with proportional odds did not hold (**Section S1.1**) and when the cumulative logit model with proportional odds held (**Section S1.2**). Section S2 contains results for simulations comparing methods for combination selection. (PDF 3152 kb)

## Abbreviations

AKI: Acute kidney injury
AUC: Area under the receiver operating characteristic curve
h-FABP: heart-type fatty acid binding protein
IL-6: interleukin-6
IL-18: interleukin-18
NT-proBNP: N-terminal-pro-B-type natriuretic peptide
ROC: Receiver operating characteristic
TRIBE-AKI: Translational research investigating biomarker endpoints in acute kidney injury
