# Supplementary material for: Using ordinal outcomes to construct and select biomarker combinations for single-level prediction
Source: Diagn Progn Res. 2018 May 21;2:8. doi: 10.1186/s41512-018-0028-3 (PMC6460803; doi:10.1186/s41512-018-0028-3)
Supplement: Supplementary file 1 — An additional file (“Additional File 1.pdf”) contains Sections S1 and S2. Section S1 contains results for simulations comparing methods for constructing combinations when the cumulative logit model with proportional odds did not hold (Section S1.1) and when the cumulative logit model with proportional odds held (Section S1.2). Section S2 contains results for simulations comparing methods for combination selection. (PDF 3152 kb) [file 41512_2018_28_MOESM1_ESM.pdf]

Supplementary Material for "Using Ordinal Outcomes to Construct and Select Biomarker Combinations for Single-level Prediction"

Allison Meisner[1], Chirag R. Parikh[2,3], and Kathleen F. Kerr[4]

[1]Department of Biostatistics, Johns Hopkins Bloomberg School of Public Health, Baltimore, Maryland

[2]Program of Applied Translational Research, Department of Medicine, Yale School of Medicine, New Haven, Connecticut

[3]Department of Internal Medicine, Veterans Affairs Medical Center, West Haven, Connecticut

[4]Department of Biostatistics, University of Washington, Seattle, Washington

ameisne1@jhu.edu

S1 Constructing Combinations

S1.1 Cumulative Logit Model with Proportional Odds Did Not Hold

For the simulations where the cumulative logit model with proportional odds did not hold, the biomarkers had multivariate normal distributions conditional on D. We considered four different sets of covariance matrices for these distributions, and we call these sets Σ_X where Σ_X may be Σ_1, Σ_2, Σ_3, or Σ_4. These four sets of matrices are defined below.

For $K = 3$, we have:

- Σ_1
  - ◇ For $D = 1$, $D = 2$, and $D = 3$: $2\begin{pmatrix} 1 & 0 \\ 0 & 1 \end{pmatrix}$
- Σ_2
  - ◇ For $D = 1$, $D = 2$, and $D = 3$: $\begin{pmatrix} 2 & 1 \\ 1 & 2 \end{pmatrix}$
- Σ_3
  - ◇ For $D = 1$: $2\begin{pmatrix} 1 & 0 \\ 0 & 1 \end{pmatrix}$
  - ◇ For $D = 2$ and $D = 3$: $2\begin{pmatrix} 1 & 0.8 \\ 0.8 & 1 \end{pmatrix}$
- Σ_4

- $\diamond$ For $D = 1$ and $D = 2$: $2\begin{pmatrix} 1 & 0 \\ 0 & 1 \end{pmatrix}$
- $\diamond$ For $D = 3$: $2\begin{pmatrix} 1 & 0.8 \\ 0.8 & 1 \end{pmatrix}$

For $K = 5$, we have:

- Σ_1
  - $\diamond$ For $D = 1$, $D = 2$, $D = 3$, $D = 4$, and $D = 5$: $2\begin{pmatrix} 1 & 0 \\ 0 & 1 \end{pmatrix}$
- Σ_2
  - $\diamond$ For $D = 1$, $D = 2$, $D = 3$, $D = 4$, and $D = 5$: $\begin{pmatrix} 2 & 1 \\ 1 & 2 \end{pmatrix}$
- Σ_3
  - $\diamond$ For $D = 1$, $D = 2$, and $D = 3$: $2\begin{pmatrix} 1 & 0 \\ 0 & 1 \end{pmatrix}$
  - $\diamond$ For $D = 4$ and $D = 5$: $2\begin{pmatrix} 1 & 0.8 \\ 0.8 & 1 \end{pmatrix}$
- Σ_4
  - $\diamond$ For $D = 1$, $D = 2$, $D = 3$, and $D = 4$: $2\begin{pmatrix} 1 & 0 \\ 0 & 1 \end{pmatrix}$

$\diamond$ For $D = 5$: $2\begin{pmatrix} 1 & 0.8 \\ 0.8 & 1 \end{pmatrix}$

The results for $\Sigma_X = \Sigma_1$ were presented in the paper.

S1.1.1 $K = 3$

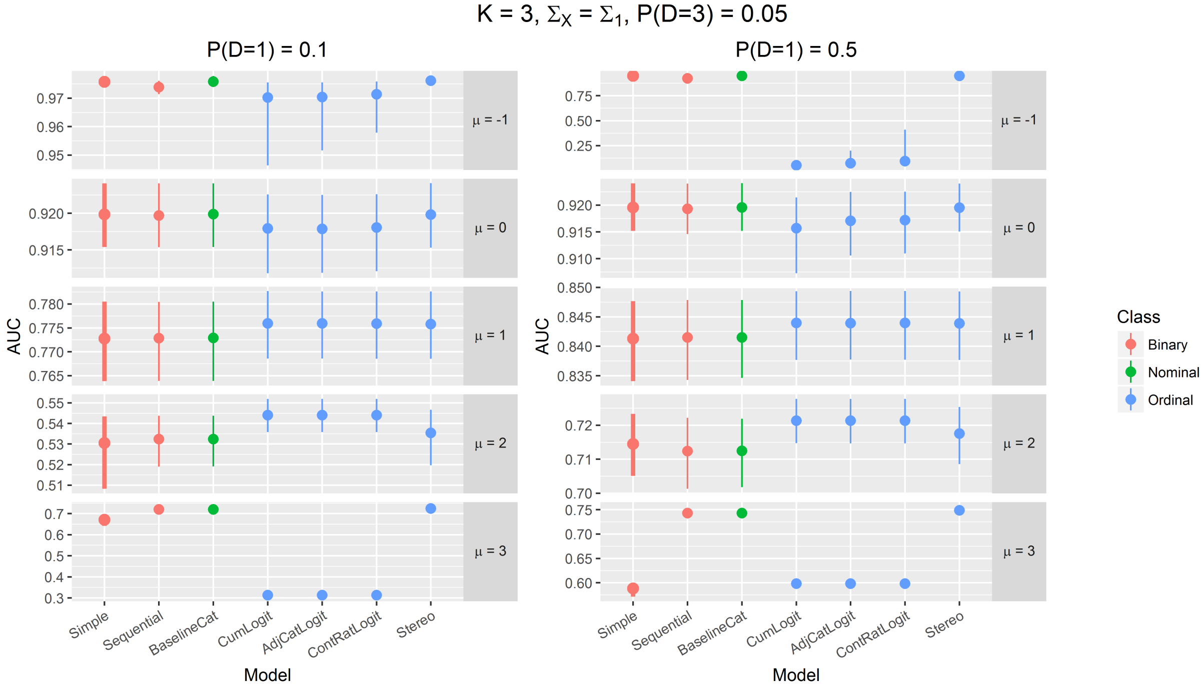

Figure S1: Simulation results for $K = 3$, $n = 400$, $P(D = 3) = 0.05$, and $\Sigma_X = \Sigma_1$ when the cumulative logit model with proportional odds did not hold. Each plot presents the median and interquartile range of the AUCs for $D = K$ vs. $D < K$ in the test data for the combinations fitted by each modeling strategy, which are given on the x-axis. The results are presented by $P(D = 1)$ (columns) and $\boldsymbol{\mu}$ (rows). The simple approach is indicated by a slightly thicker line and larger point.

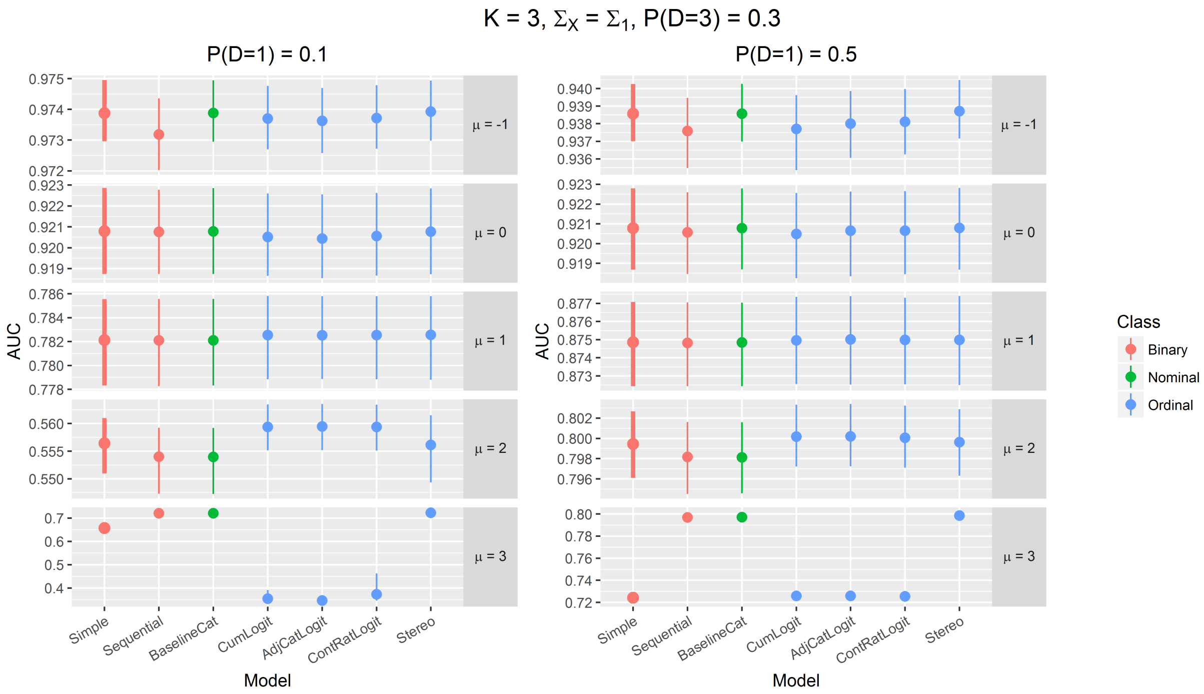

Figure S2: Simulation results for $K = 3$, $n = 400$, $P(D = 3) = 0.3$, and $\Sigma_X = \Sigma_1$ when the cumulative logit model with proportional odds did not hold. Each plot presents the median and interquartile range of the AUCs for $D = K$ vs. $D < K$ in the test data for the combinations fitted by each modeling strategy, which are given on the x-axis. The results are presented by $P(D = 1)$ (columns) and $\boldsymbol{\mu}$ (rows). The simple approach is indicated by a slightly thicker line and larger point.

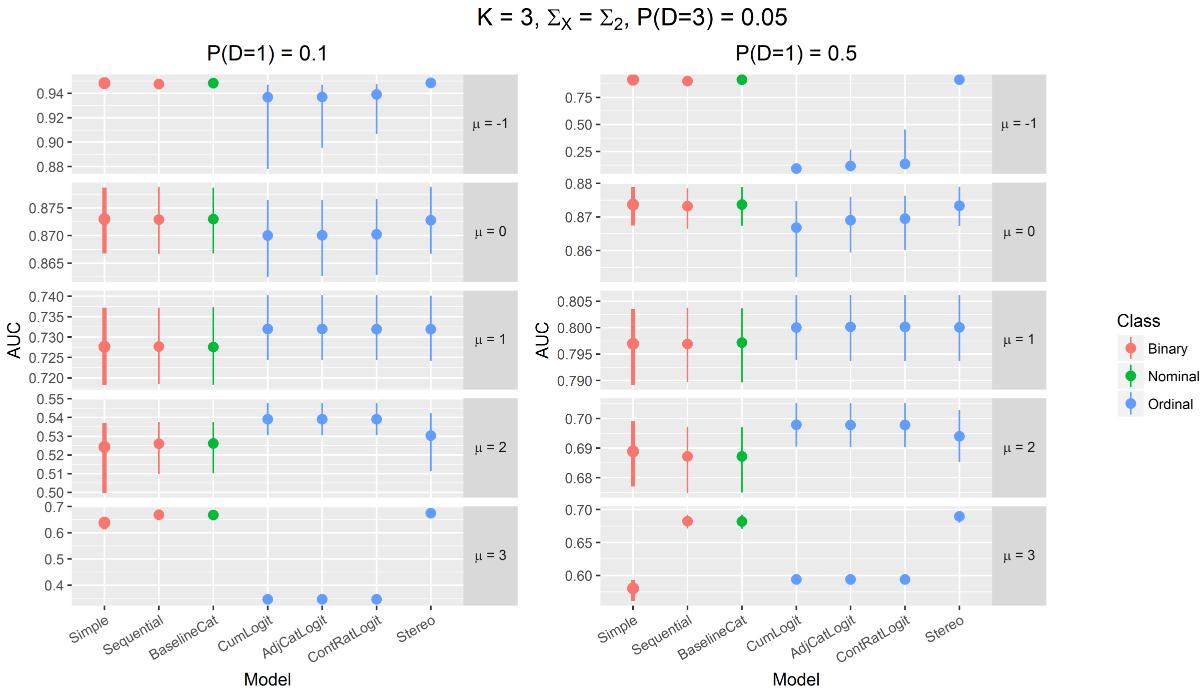

Figure S3: Simulation results for $K = 3$, $n = 400$, $P(D = 3) = 0.05$, and $\Sigma_X = \Sigma_2$ when the cumulative logit model with proportional odds did not hold. Each plot presents the median and interquartile range of the AUCs for $D = K$ vs. $D < K$ in the test data for the combinations fitted by each modeling strategy, which are given on the x-axis. The results are presented by $P(D = 1)$ (columns) and $\boldsymbol{\mu}$ (rows). The simple approach is indicated by a slightly thicker line and larger point.

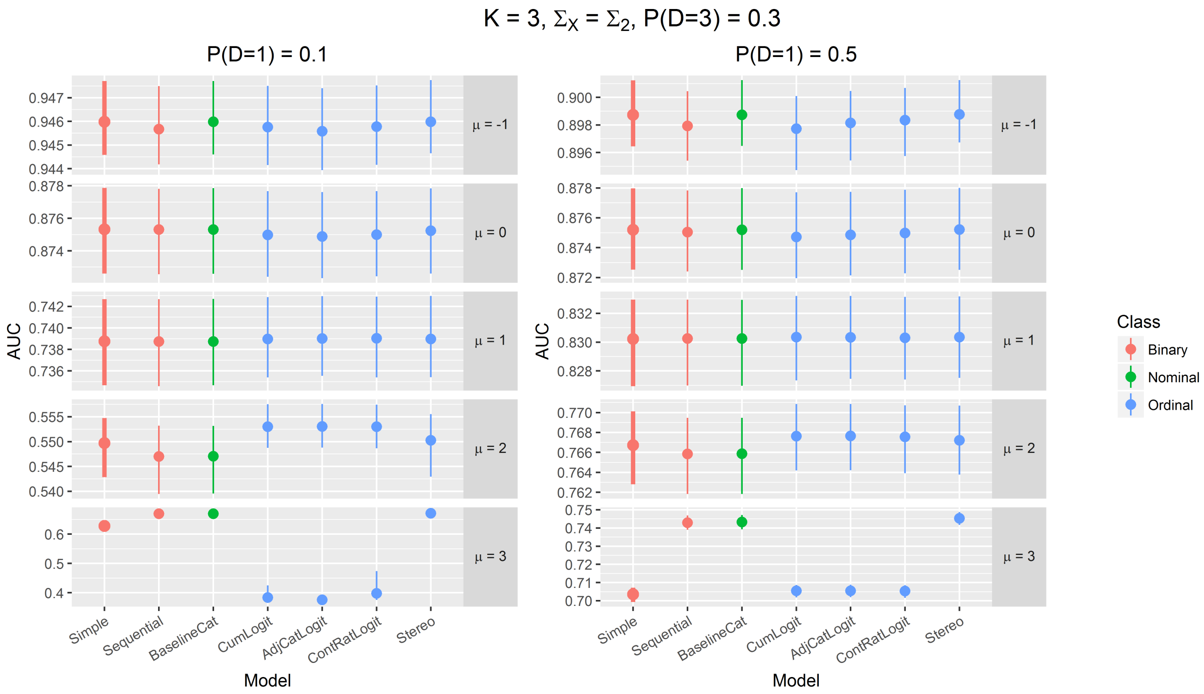

Figure S4: Simulation results for $K = 3$, $n = 400$, $P(D = 3) = 0.3$, and $\Sigma_X = \Sigma_2$ when the cumulative logit model with proportional odds did not hold. Each plot presents the median and interquartile range of the AUCs for $D = K$ vs. $D < K$ in the test data for the combinations fitted by each modeling strategy, which are given on the x-axis. The results are presented by $P(D = 1)$ (columns) and $\boldsymbol{\mu}$ (rows). The simple approach is indicated by a slightly thicker line and larger point.

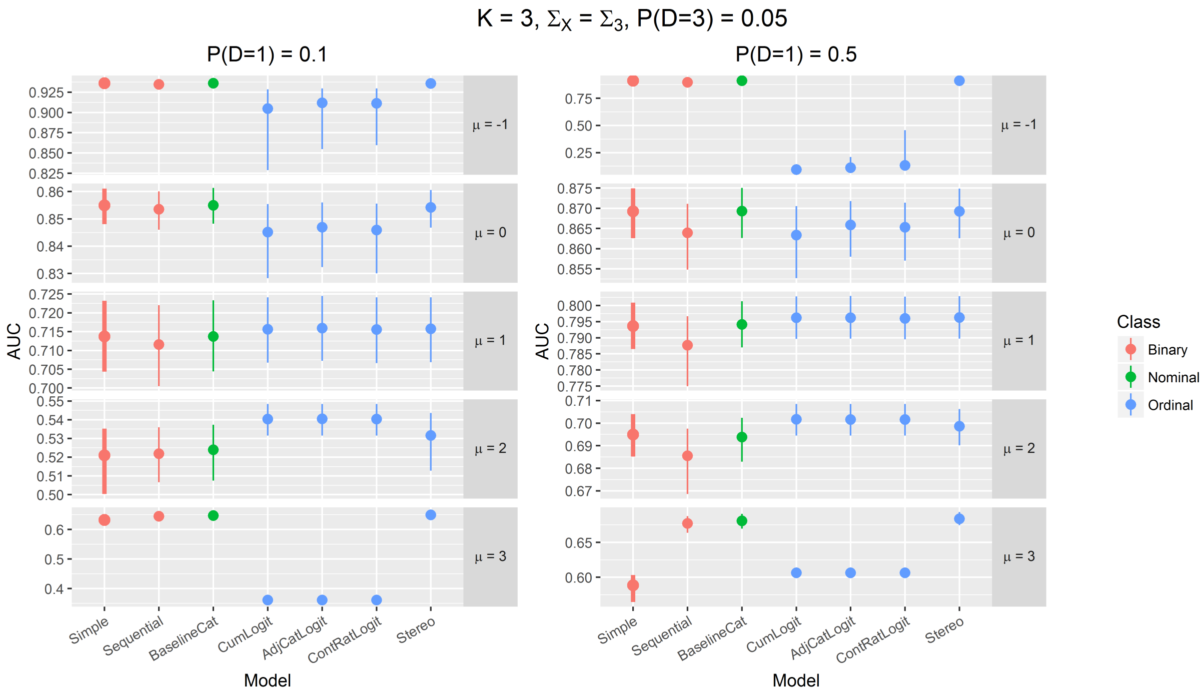

Figure S5: Simulation results for $K = 3$, $n = 400$, $P(D = 3) = 0.05$, and $\Sigma_X = \Sigma_3$ when the cumulative logit model with proportional odds did not hold. Each plot presents the median and interquartile range of the AUCs for $D = K$ vs. $D < K$ in the test data for the combinations fitted by each modeling strategy, which are given on the x-axis. The results are presented by $P(D = 1)$ (columns) and $\boldsymbol{\mu}$ (rows). The simple approach is indicated by a slightly thicker line and larger point.

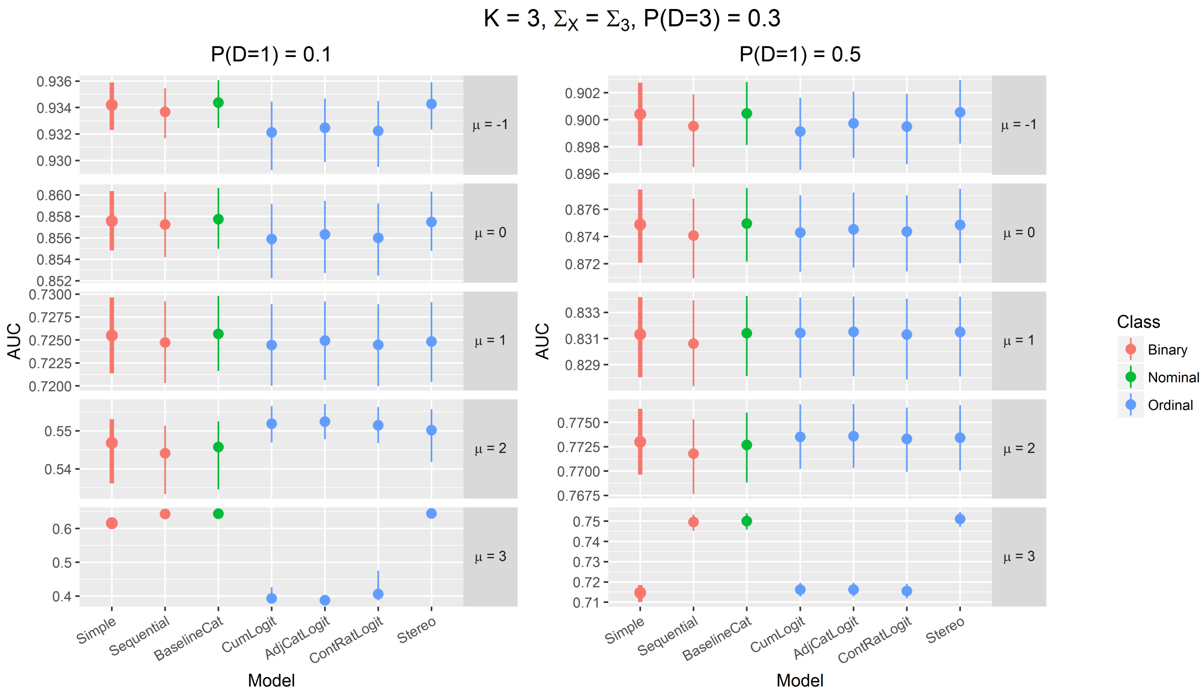

Figure S6: Simulation results for $K = 3$, $n = 400$, $P(D = 3) = 0.3$, and $\Sigma_X = \Sigma_3$ when the cumulative logit model with proportional odds did not hold. Each plot presents the median and interquartile range of the AUCs for $D = K$ vs. $D < K$ in the test data for the combinations fitted by each modeling strategy, which are given on the x-axis. The results are presented by $P(D = 1)$ (columns) and $\boldsymbol{\mu}$ (rows). The simple approach is indicated by a slightly thicker line and larger point.

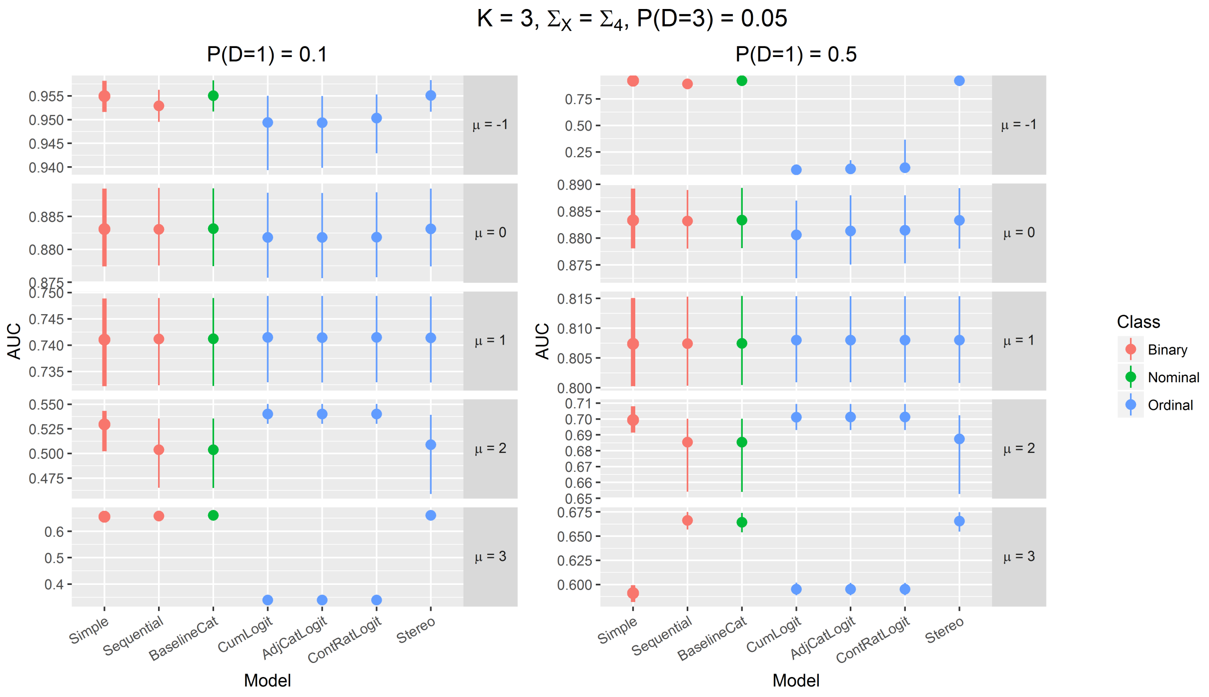

Figure S7: Simulation results for $K = 3$, $n = 400$, $P(D = 3) = 0.05$, and $\Sigma_X = \Sigma_4$ when the cumulative logit model with proportional odds did not hold. Each plot presents the median and interquartile range of the AUCs for $D = K$ vs. $D < K$ in the test data for the combinations fitted by each modeling strategy, which are given on the x-axis. The results are presented by $P(D = 1)$ (columns) and $\boldsymbol{\mu}$ (rows). The simple approach is indicated by a slightly thicker line and larger point.

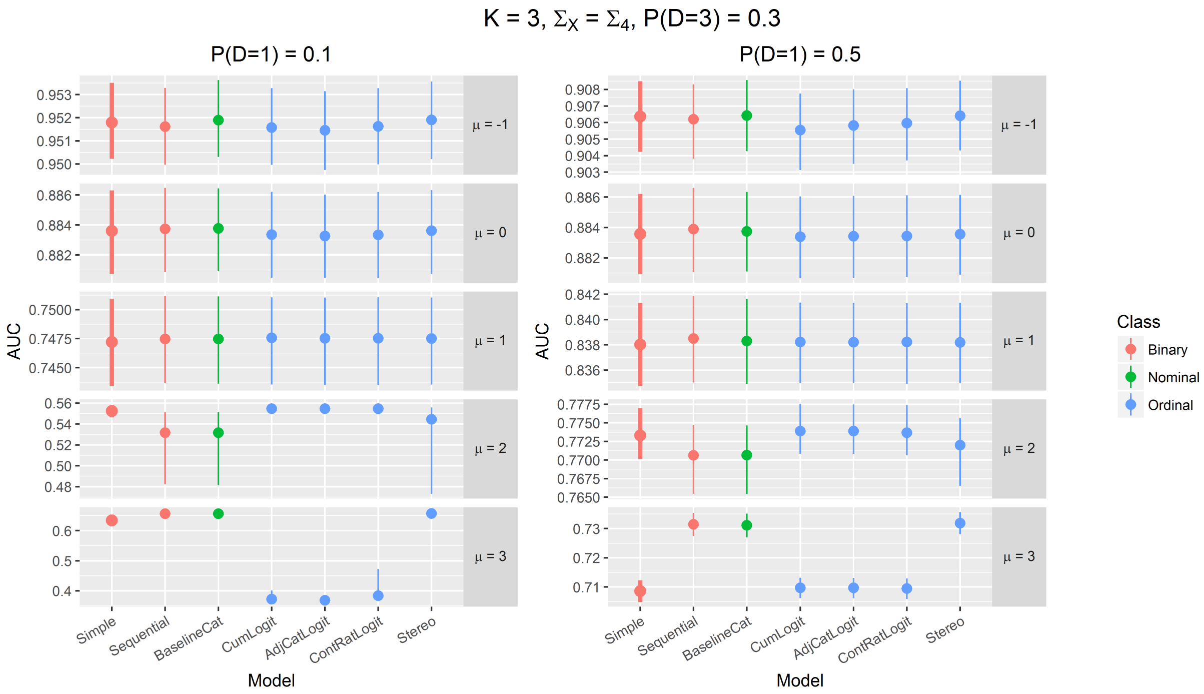

Figure S8: Simulation results for $K = 3$, $n = 400$, $P(D = 3) = 0.3$, and $\Sigma_X = \Sigma_4$ when the cumulative logit model with proportional odds did not hold. Each plot presents the median and interquartile range of the AUCs for $D = K$ vs. $D < K$ in the test data for the combinations fitted by each modeling strategy, which are given on the x-axis. The results are presented by $P(D = 1)$ (columns) and $\boldsymbol{\mu}$ (rows). The simple approach is indicated by a slightly thicker line and larger point.

S1.1.2 $K = 5$

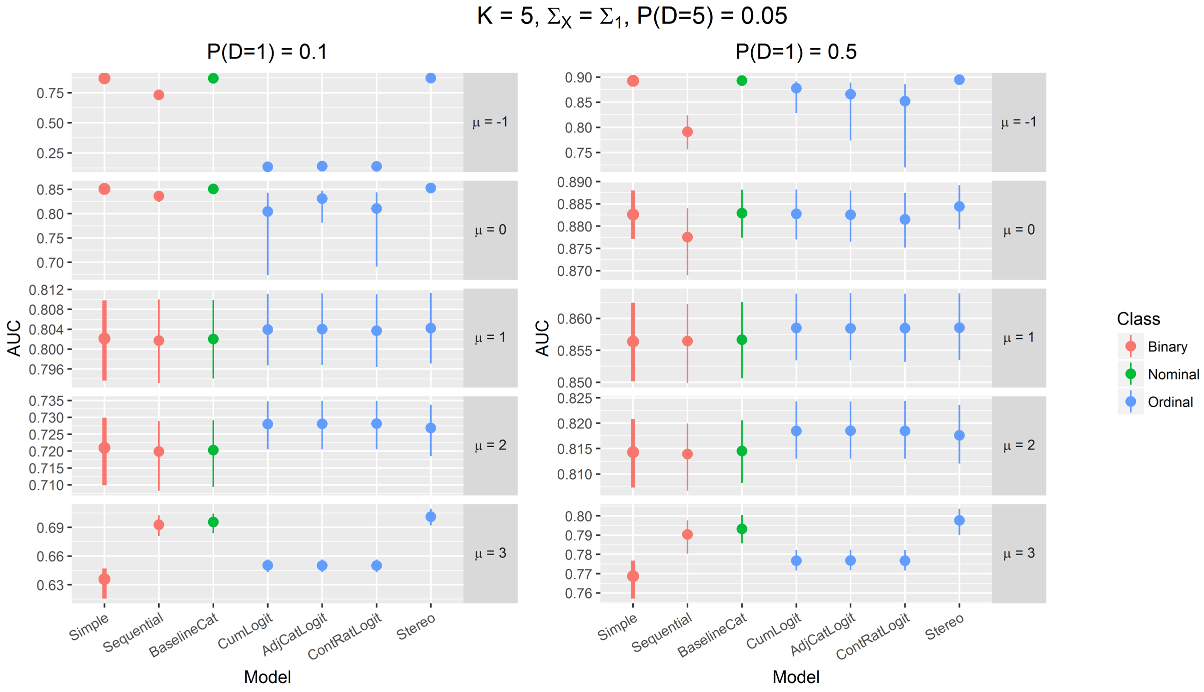

Figure S9: Simulation results for $K = 5$, $n = 400$, $P(D = 5) = 0.05$, and $\Sigma_X = \Sigma_1$ when the cumulative logit model with proportional odds did not hold. Each plot presents the median and interquartile range of the AUCs for $D = K$ vs. $D < K$ in the test data for the combinations fitted by each modeling strategy, which are given on the x-axis. The results are presented by $P(D = 1)$ (columns) and $\boldsymbol{\mu}$ (rows). The simple approach is indicated by a slightly thicker line and larger point.

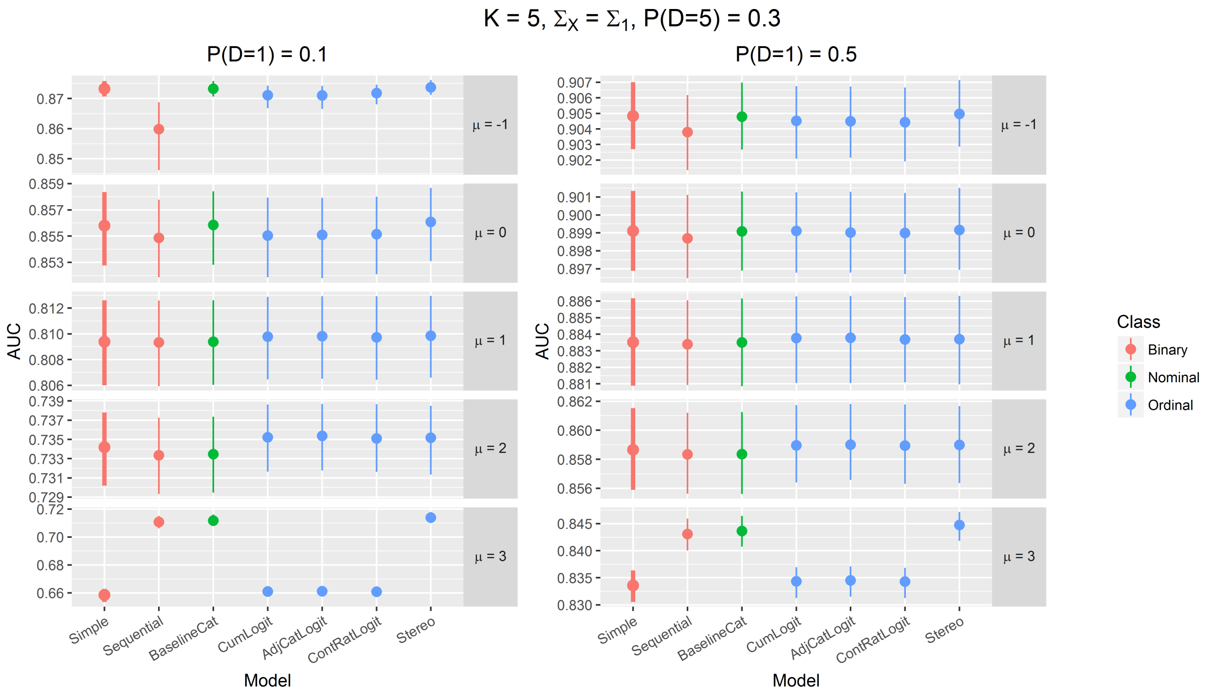

Figure S10: Simulation results for $K = 5$, $n = 400$, $P(D = 5) = 0.3$, and $\Sigma_X = \Sigma_1$ when the cumulative logit model with proportional odds did not hold. Each plot presents the median and interquartile range of the AUCs for $D = K$ vs. $D < K$ in the test data for the combinations fitted by each modeling strategy, which are given on the x-axis. The results are presented by $P(D = 1)$ (columns) and $\boldsymbol{\mu}$ (rows). The simple approach is indicated by a slightly thicker line and larger point.

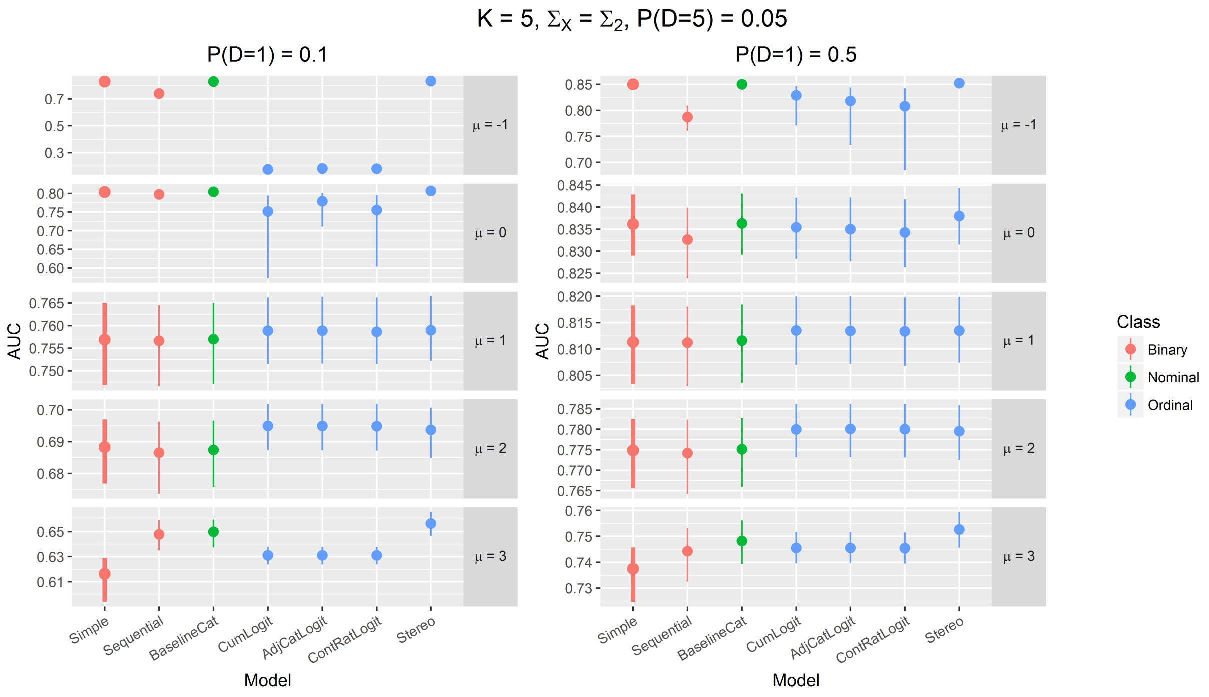

Figure S11: Simulation results for $K = 5$, $n = 400$, $P(D = 5) = 0.05$, and $\Sigma_X = \Sigma_2$ when the cumulative logit model with proportional odds did not hold. Each plot presents the median and interquartile range of the AUCs for $D = K$ vs. $D < K$ in the test data for the combinations fitted by each modeling strategy, which are given on the x-axis. The results are presented by $P(D = 1)$ (columns) and $\boldsymbol{\mu}$ (rows). The simple approach is indicated by a slightly thicker line and larger point.

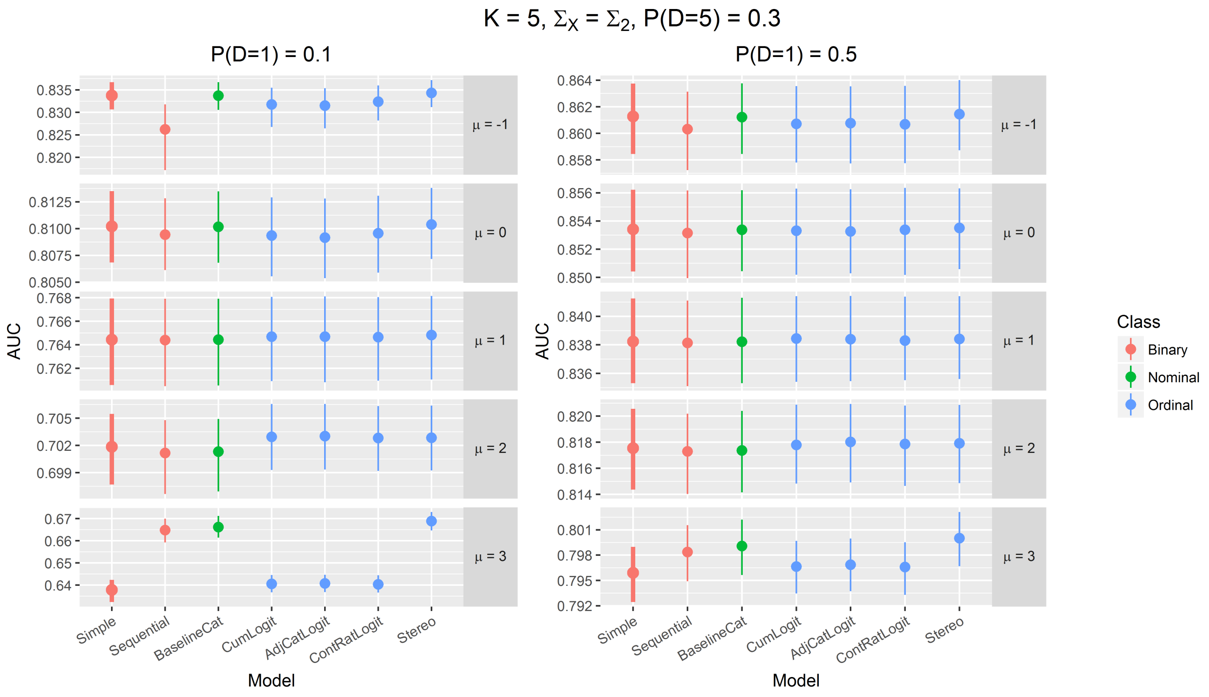

Figure S12: Simulation results for $K = 5$, $n = 400$, $P(D = 5) = 0.3$, and $\Sigma_X = \Sigma_2$ when the cumulative logit model with proportional odds did not hold. Each plot presents the median and interquartile range of the AUCs for $D = K$ vs. $D < K$ in the test data for the combinations fitted by each modeling strategy, which are given on the x-axis. The results are presented by $P(D = 1)$ (columns) and $\boldsymbol{\mu}$ (rows). The simple approach is indicated by a slightly thicker line and larger point.

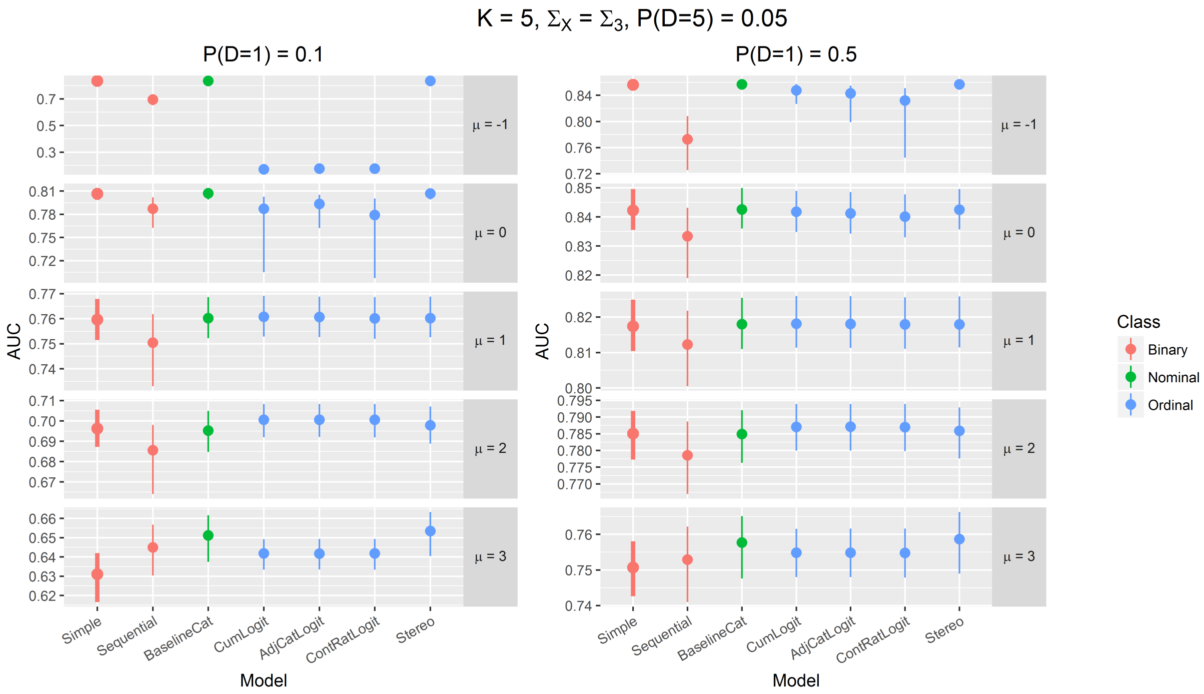

Figure S13: Simulation results for $K = 5$, $n = 400$, $P(D = 5) = 0.05$, and $\Sigma_X = \Sigma_3$ when the cumulative logit model with proportional odds did not hold. Each plot presents the median and interquartile range of the AUCs for $D = K$ vs. $D < K$ in the test data for the combinations fitted by each modeling strategy, which are given on the x-axis. The results are presented by $P(D = 1)$ (columns) and $\boldsymbol{\mu}$ (rows). The simple approach is indicated by a slightly thicker line and larger point.

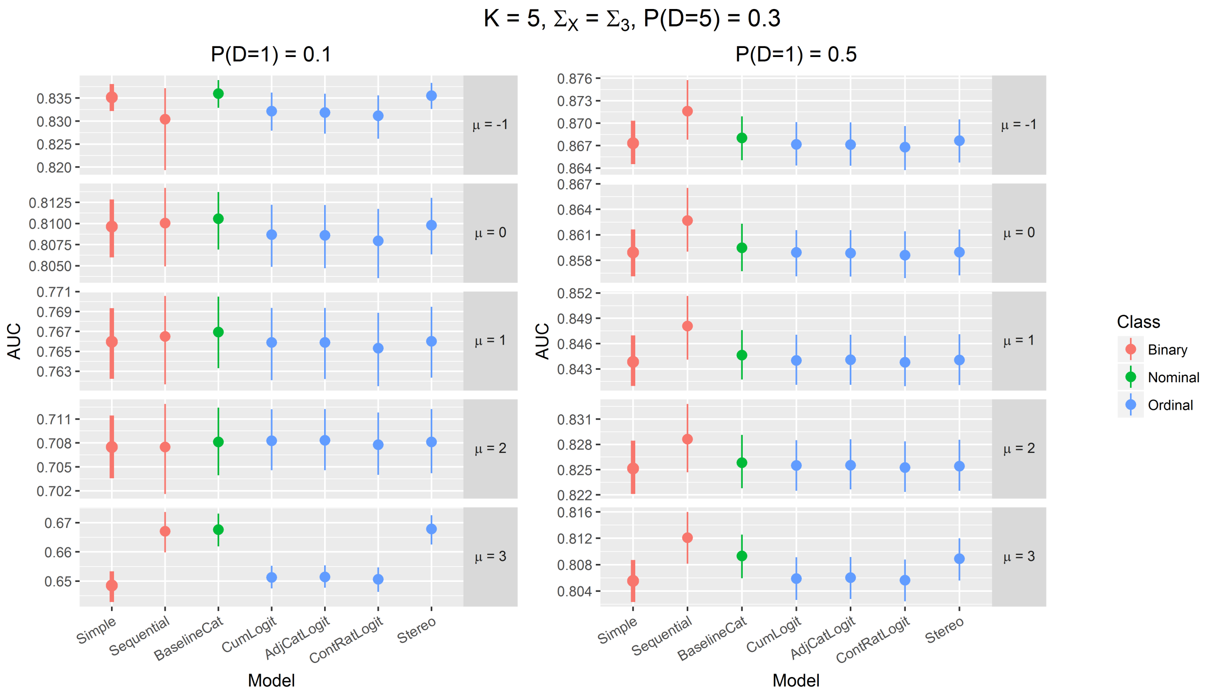

Figure S14: Simulation results for $K = 5$, $n = 400$, $P(D = 5) = 0.3$, and $\Sigma_X = \Sigma_3$ when the cumulative logit model with proportional odds did not hold. Each plot presents the median and interquartile range of the AUCs for $D = K$ vs. $D < K$ in the test data for the combinations fitted by each modeling strategy, which are given on the x-axis. The results are presented by $P(D = 1)$ (columns) and $\boldsymbol{\mu}$ (rows). The simple approach is indicated by a slightly thicker line and larger point.

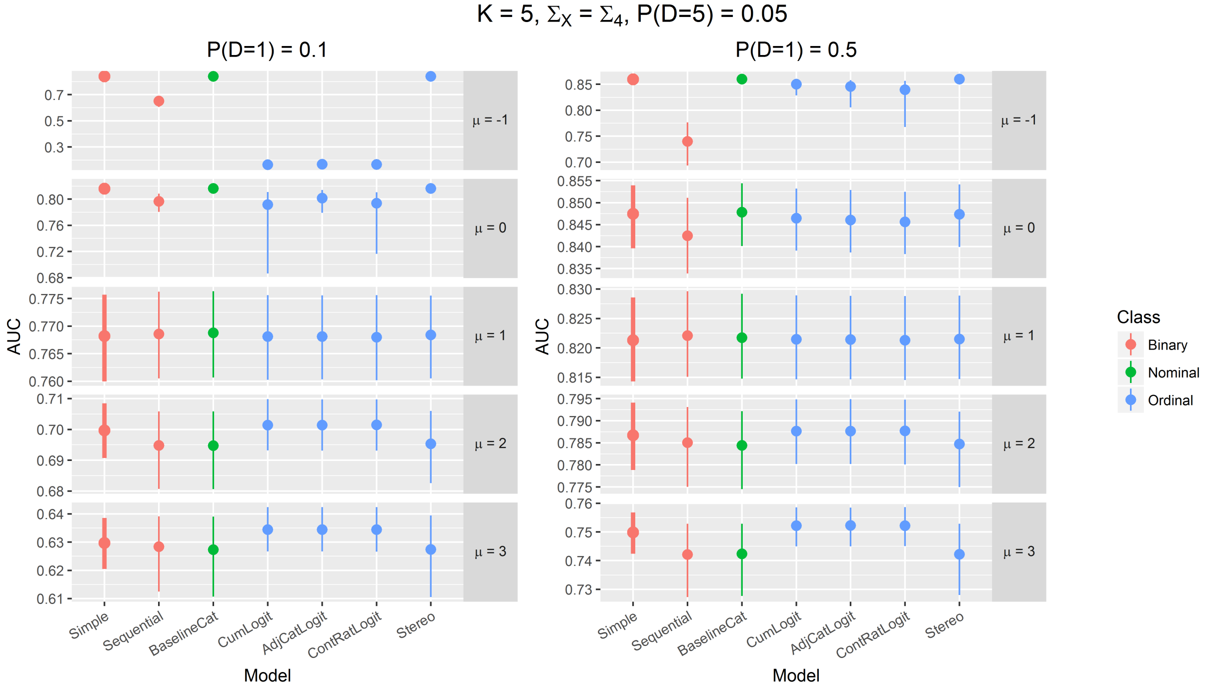

Figure S15: Simulation results for $K = 5$, $n = 400$, $P(D = 5) = 0.05$, and $\Sigma_X = \Sigma_4$ when the cumulative logit model with proportional odds did not hold. Each plot presents the median and interquartile range of the AUCs for $D = K$ vs. $D < K$ in the test data for the combinations fitted by each modeling strategy, which are given on the x-axis. The results are presented by $P(D = 1)$ (columns) and $\boldsymbol{\mu}$ (rows). The simple approach is indicated by a slightly thicker line and larger point.

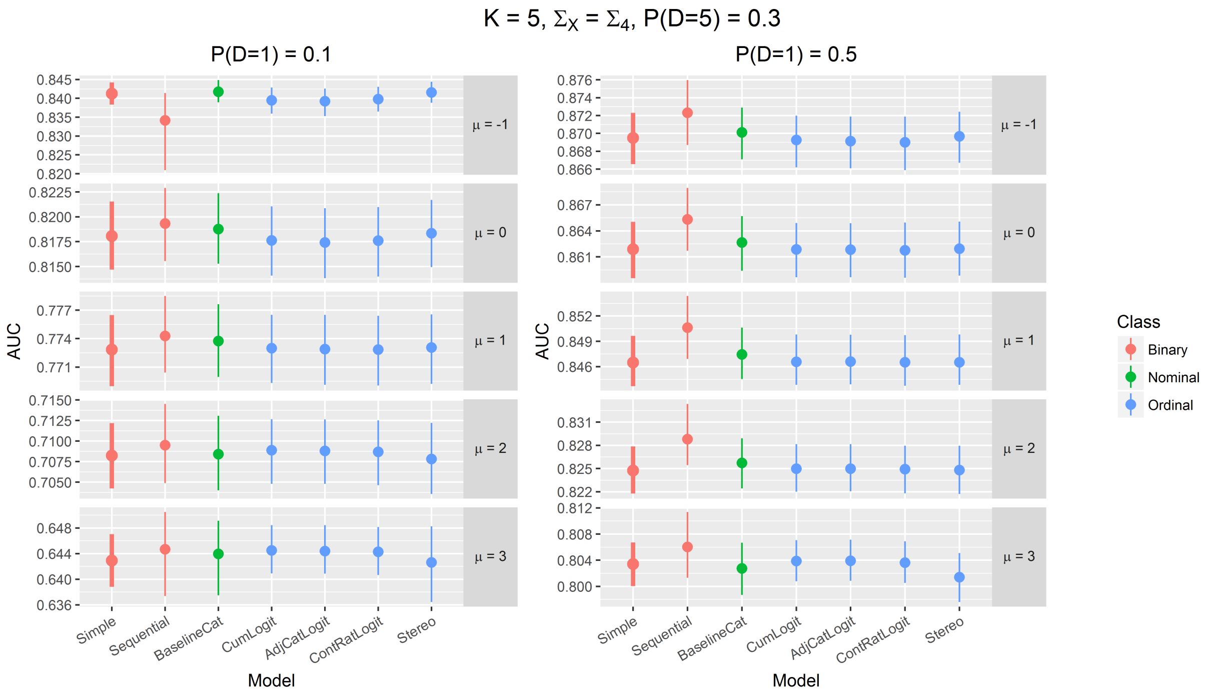

Figure S16: Simulation results for $K = 5$, $n = 400$, $P(D = 5) = 0.3$, and $\Sigma_X = \Sigma_4$ when the cumulative logit model with proportional odds did not hold. Each plot presents the median and interquartile range of the AUCs for $D = K$ vs. $D < K$ in the test data for the combinations fitted by each modeling strategy, which are given on the x-axis. The results are presented by $P(D = 1)$ (columns) and $\boldsymbol{\mu}$ (rows). The simple approach is indicated by a slightly thicker line and larger point.

S1.2 Cumulative Logit Model with Proportional Odds Held

S1.2.1 $K = 3$

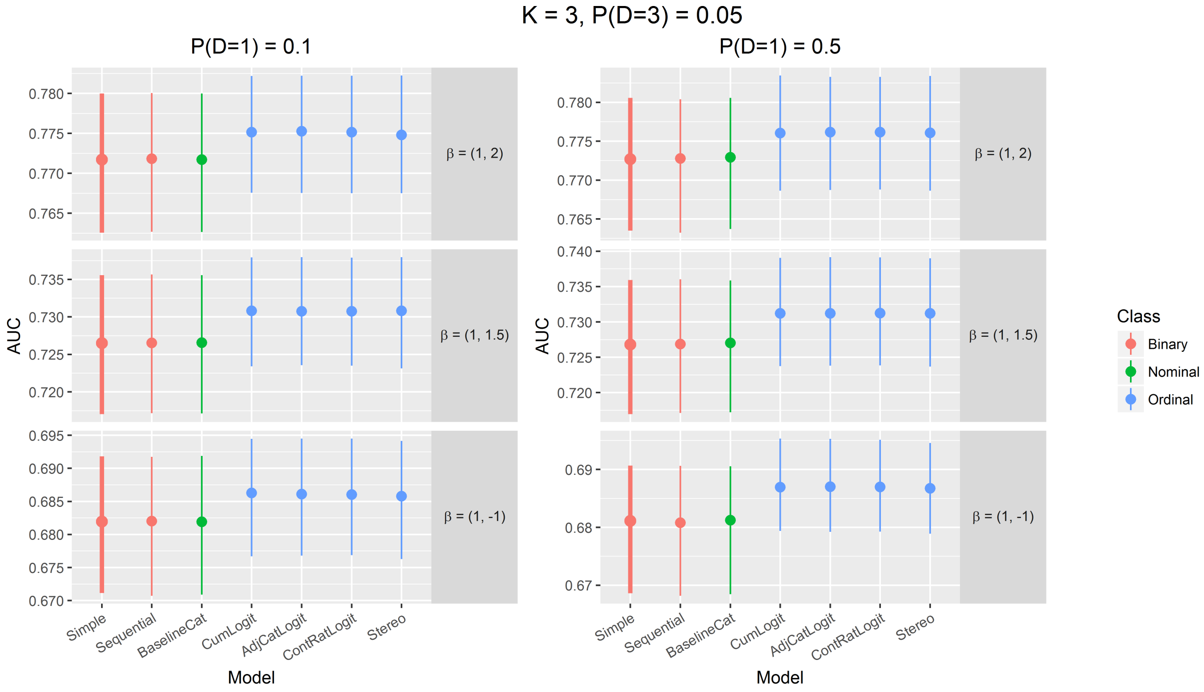

Figure S17: Simulation results for $K = 3$, $n = 400$, and $P(D = 3) = 0.05$ when the cumulative logit model with proportional odds held. Each plot presents the median and interquartile range of the AUCs for $D = K$ vs. $D < K$ in the test data for the combinations fitted by each modeling strategy, which are given on the x-axis. The results are presented by $P(D = 1)$ (columns) and $\boldsymbol{\mu}$ (rows). The simple approach is indicated by a slightly thicker line and larger point.

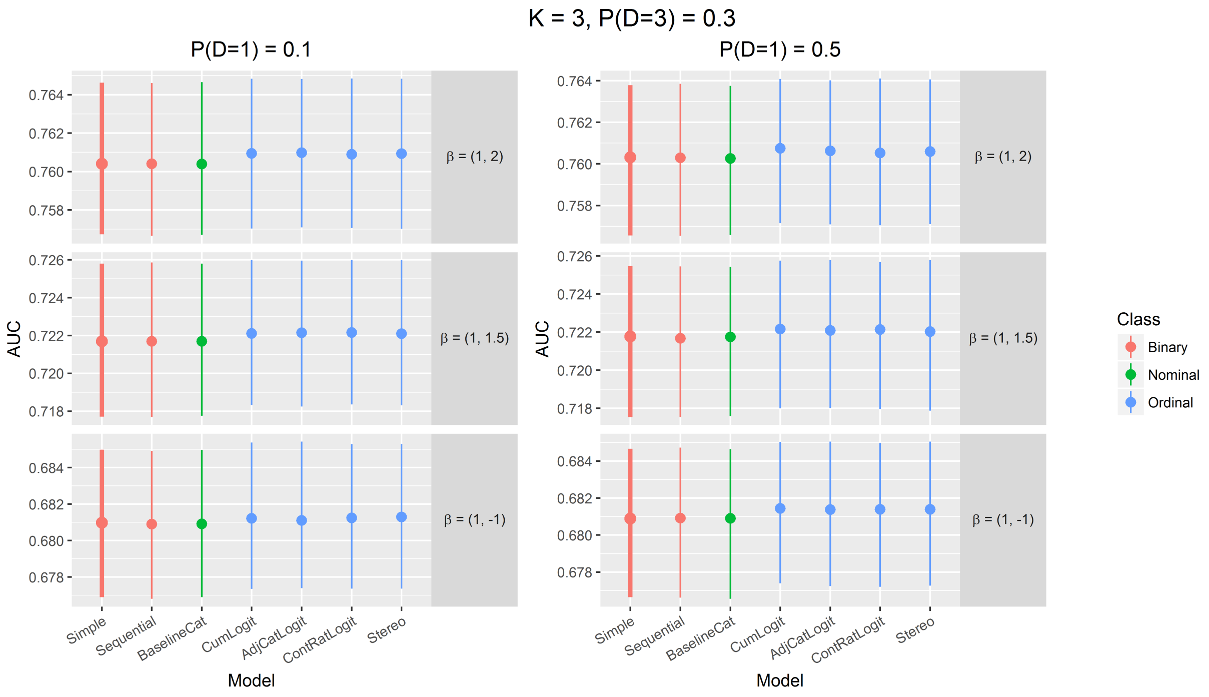

Figure S18: Simulation results for $K = 3$, $n = 400$, and $P(D = 3) = 0.3$ when the cumulative logit model with proportional odds held. Each plot presents the median and interquartile range of the AUCs for $D = K$ vs. $D < K$ in the test data for the combinations fitted by each modeling strategy, which are given on the x-axis. The results are presented by $P(D = 1)$ (columns) and $\boldsymbol{\mu}$ (rows). The simple approach is indicated by a slightly thicker line and larger point.

S1.2.2 $K = 5$

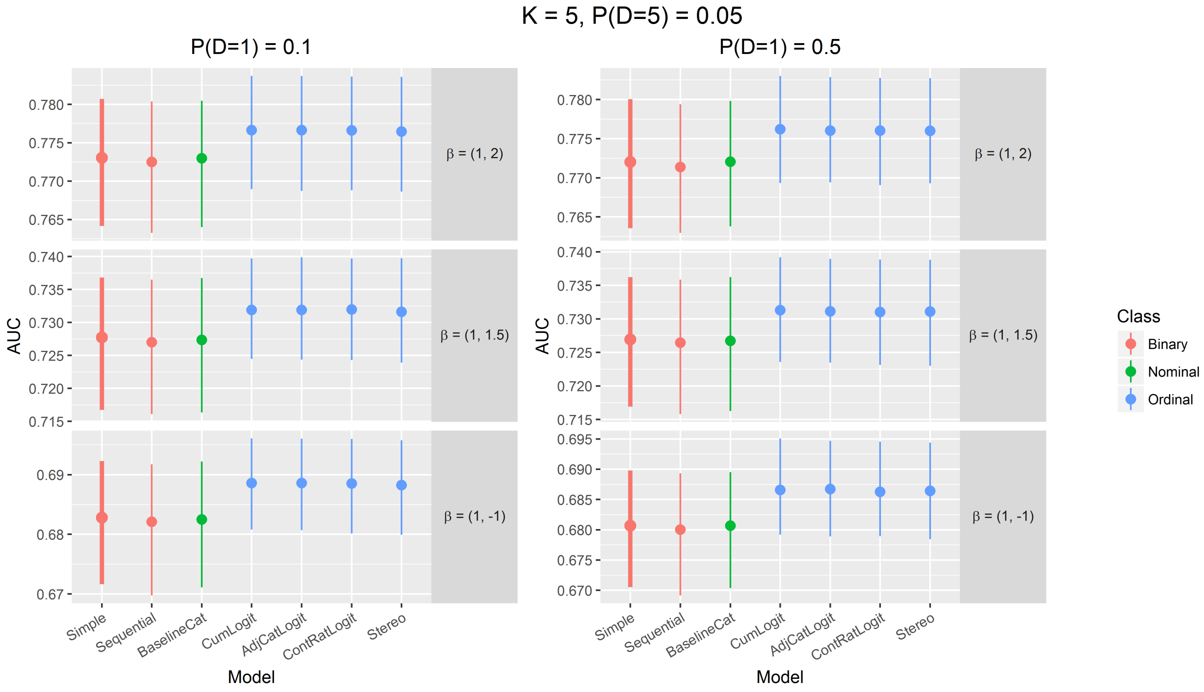

Figure S19: Simulation results for $K = 5$, $n = 400$, and $P(D = 5) = 0.05$ when the cumulative logit model with proportional odds held. Each plot presents the median and interquartile range of the AUCs for $D = K$ vs. $D < K$ in the test data for the combinations fitted by each modeling strategy, which are given on the x-axis. The results are presented by $P(D = 1)$ (columns) and $\boldsymbol{\mu}$ (rows). The simple approach is indicated by a slightly thicker line and larger point.

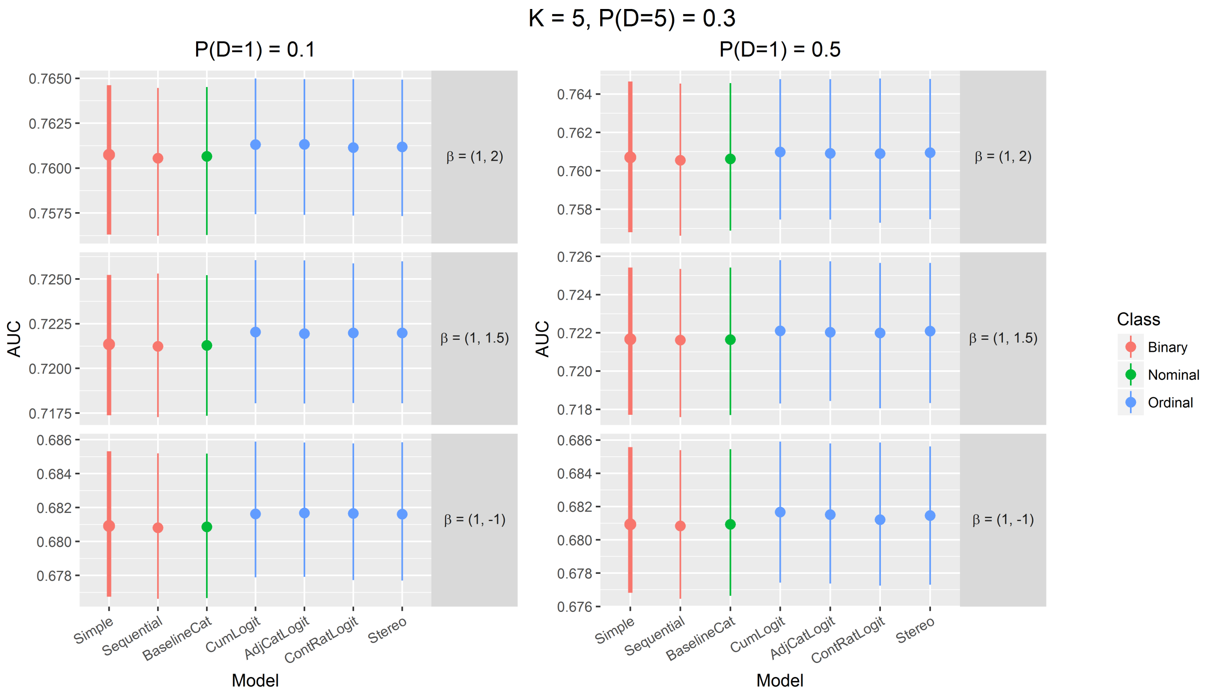

Figure S20: Simulation results for $K = 5$, $n = 400$, and $P(D = 5) = 0.3$ when the cumulative logit model with proportional odds held. Each plot presents the median and interquartile range of the AUCs for $D = K$ vs. $D < K$ in the test data for the combinations fitted by each modeling strategy, which are given on the x-axis. The results are presented by $P(D = 1)$ (columns) and $\boldsymbol{\mu}$ (rows). The simple approach is indicated by a slightly thicker line and larger point.

S2 Combination Selection

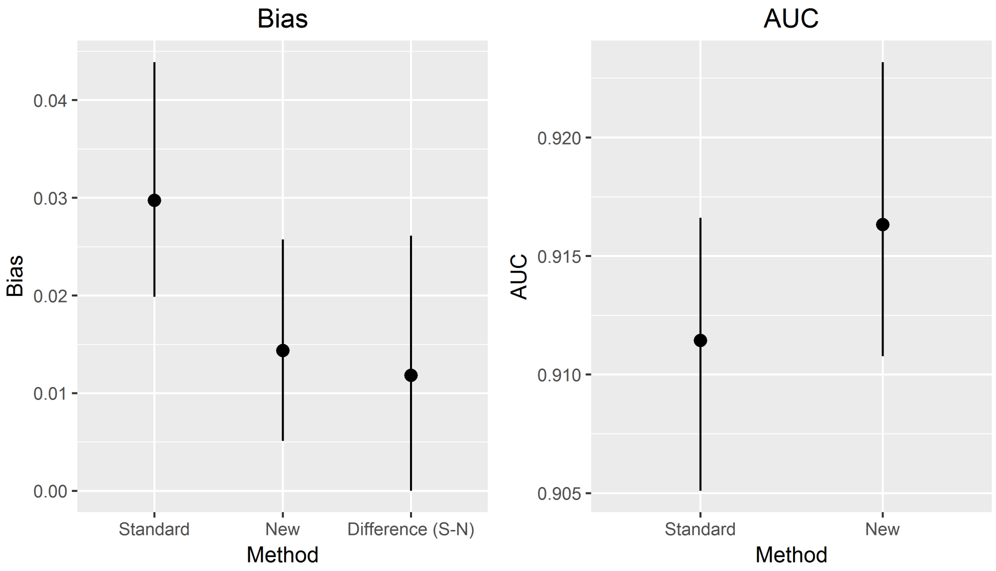

Figure S21: Results for the proposed combination selection method for Example 1. The plot on the left gives the median and interquartile range of the estimated model selection bias for the combinations selected by the two approaches (the standard approach and the new approach) and the difference in the estimated bias between the two approaches. The plot on the right gives the median and interquartile range of the AUC for $D = 3$ vs. $D < 3$ in test data for the combinations selected by the two approaches.

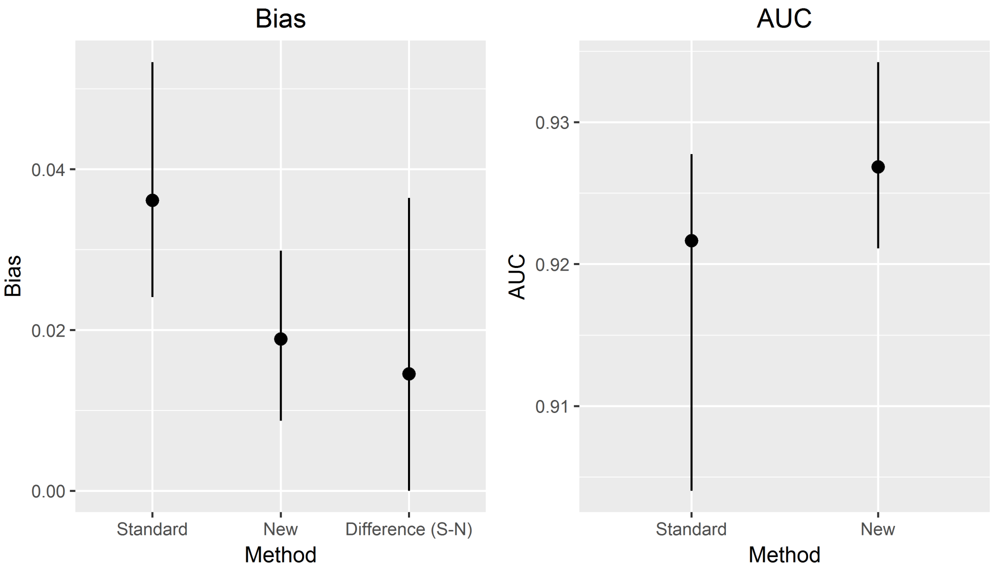

Figure S22: Results for the proposed combination selection method for Example 2. The plot on the left gives the median and interquartile range of the estimated model selection bias for the combinations selected by the two approaches (the standard approach and the new approach) and the difference in the estimated bias between the two approaches. The plot on the right gives the median and interquartile range of the AUC for $D = 3$ vs. $D < 3$ in test data for the combinations selected by the two approaches.

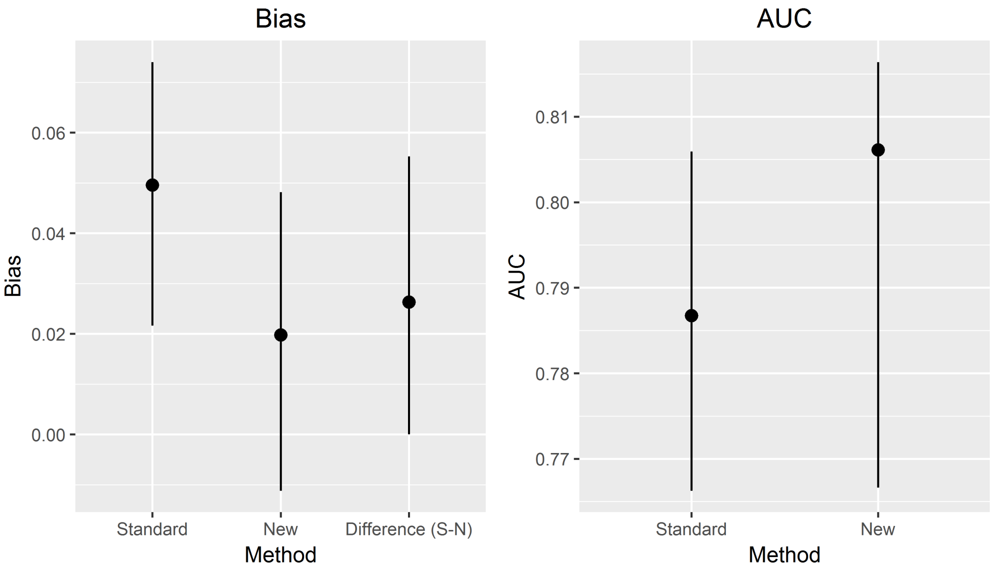

Figure S23: Results for the proposed combination selection method for Example 3. The plot on the left gives the median and interquartile range of the estimated model selection bias for the combinations selected by the two approaches (the standard approach and the new approach) and the difference in the estimated bias between the two approaches. The plot on the right gives the median and interquartile range of the AUC for $D = 3$ vs. $D < 3$ in test data for the combinations selected by the two approaches.

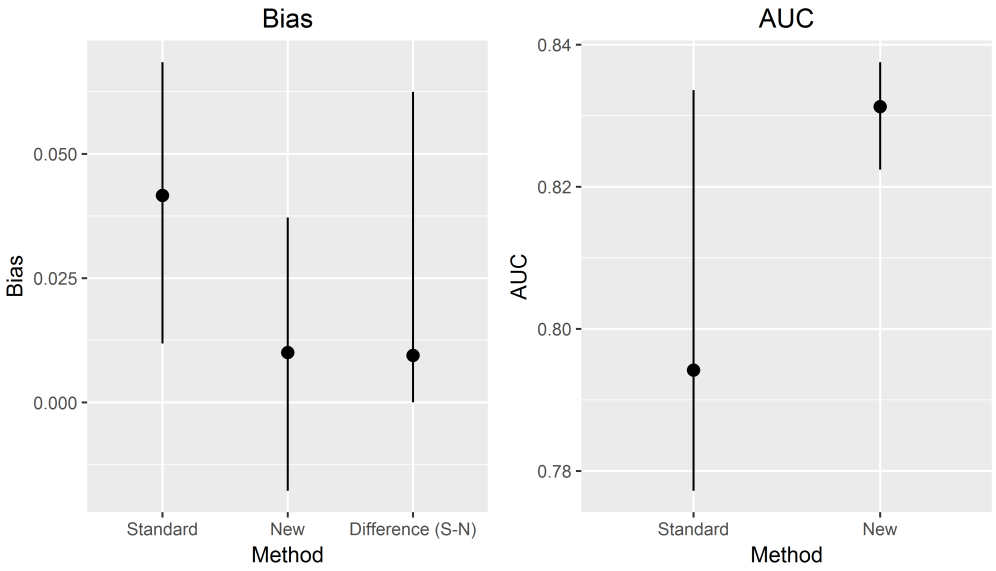

Figure S24: Results for the proposed combination selection method for Example 4. The plot on the left gives the median and interquartile range of the estimated model selection bias for the combinations selected by the two approaches (the standard approach and the new approach) and the difference in the estimated bias between the two approaches. The plot on the right gives the median and interquartile range of the AUC for $D = 3$ vs. $D < 3$ in test data for the combinations selected by the two approaches.

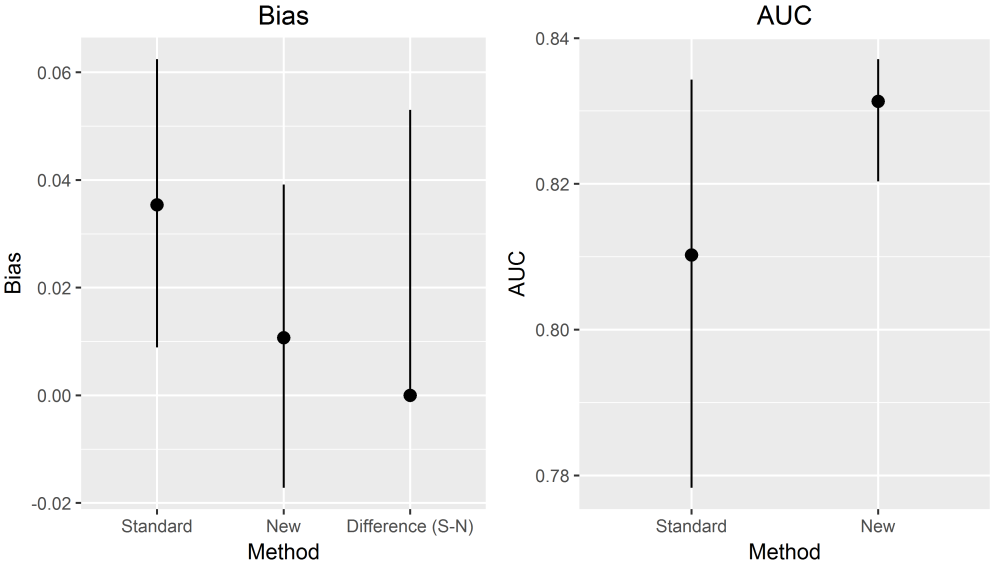

Figure S25: Results for the proposed combination selection method for Example 5. The plot on the left gives the median and interquartile range of the estimated model selection bias for the combinations selected by the two approaches (the standard approach and the new approach) and the difference in the estimated bias between the two approaches. The plot on the right gives the median and interquartile range of the AUC for $D = 3$ vs. $D < 3$ in test data for the combinations selected by the two approaches.
